# Supplementary material for: Quantitative whole-tissue 3D imaging reveals bacteria in close association with mouse jejunum mucosa
Source: NPJ Biofilms Microbiomes. 2023 Sep 7;9:64. doi: 10.1038/s41522-023-00423-2 (PMC10485000; doi:10.1038/s41522-023-00423-2)
Supplement: Supplementary file 1 — Reporting Summary [file 41522_2023_423_MOESM1_ESM.pdf]

nature research

Corresponding author(s): Rustem F. Ismagilov

Last updated by author(s): 6/23/2023

Reporting Summary

Nature Research wishes to improve the reproducibility of the work that we publish. This form provides structure for consistency and transparency in reporting. For further information on Nature Research policies, see our Editorial Policies and the Editorial Policy Checklist.

Statistics

For all statistical analyses, confirm that the following items are present in the figure legend, table legend, main text, or Methods section.

n/a	Confirmed	
☐	☒	The exact sample size (n) for each experimental group/condition, given as a discrete number and unit of measurement
☐	☒	A statement on whether measurements were taken from distinct samples or whether the same sample was measured repeatedly
☐	☒	The statistical test(s) used AND whether they are one- or two-sided<br>*Only common tests should be described solely by name; describe more complex techniques in the Methods section.*
☒	☐	A description of all covariates tested
☐	☒	A description of any assumptions or corrections, such as tests of normality and adjustment for multiple comparisons
☐	☒	A full description of the statistical parameters including central tendency (e.g. means) or other basic estimates (e.g. regression coefficient) AND variation (e.g. standard deviation) or associated estimates of uncertainty (e.g. confidence intervals)
☒	☐	For null hypothesis testing, the test statistic (e.g. F, t, r) with confidence intervals, effect sizes, degrees of freedom and P value noted<br>*Give P values as exact values whenever suitable.*
☒	☐	For Bayesian analysis, information on the choice of priors and Markov chain Monte Carlo settings
☒	☐	For hierarchical and complex designs, identification of the appropriate level for tests and full reporting of outcomes
☒	☐	Estimates of effect sizes (e.g. Cohen's d, Pearson's r), indicating how they were calculated

Our web collection on statistics for biologists contains articles on many of the points above.

Software and code

Policy information about availability of computer code

Data collection	All images were acquired on Zeiss LSM 880 confocal microscope running Zen 2.3 SP1 software. Digital PCR data (Fig. 2, Fig. S2) was collected using QX200 Droplet Digital System (1864002 and 1864003; Bio-Rad Laboratories, Hercules, CA, USA). RTqPCR data (Fig. S1) was collected using BioRad CFX96 qPCR instrument.
Data analysis	Image tile scans were stitched in Zen 2.3 SP1 software. The images were segmented in Imaris 9.7 software. All other data analysis was performed using custom scripts that are available at Caltech DATA at https://doi.org/10.22002/zg1d3-k3b49.

For manuscripts utilizing custom algorithms or software that are central to the research but not yet described in published literature, software must be made available to editors and reviewers. We strongly encourage code deposition in a community repository (e.g. GitHub). See the Nature Research guidelines for submitting code & software for further information.

Data

Policy information about availability of data

All manuscripts must include a data availability statement. This statement should provide the following information, where applicable:

- Accession codes, unique identifiers, or web links for publicly available datasets
- A list of figures that have associated raw data
- A description of any restrictions on data availability

All data and data analysis scripts from this publication are available at Caltech DATA at https://doi.org/10.22002/zg1d3-k3b49 or are uploaded as Supplementary Data.

Field-specific reporting

Please select the one below that is the best fit for your research. If you are not sure, read the appropriate sections before making your selection.

☒ Life sciences ☐ Behavioural & social sciences ☐ Ecological, evolutionary & environmental sciences

For a reference copy of the document with all sections, see nature.com/documents/nr-reporting-summary-flat.pdf

Life sciences study design

All studies must disclose on these points even when the disclosure is negative.

Sample size	Sample size calculation was not performed. The scope of the present manuscript was to test whether dense bacterial association with mouse jejunum mucosa was plausible. This phenomenon was detected in 1 (out of 4) malnourished mice gavaged with bacterial cocktail but in none of the malnourished controls, therefore, sample size was sufficient given the scope of the present manuscript.
Data exclusions	In Fig. 1, a-d, group averages were calculated considering mouse samples collected over days 28-30 of the experiment. Day 31 samples were excluded because a sudden change in microbiota composition (specifically, drop in E. coli load) was observed to occur on day 31 of the experiment.<br>In Fig. 4, d-f, 3 images per sample were analyzed. For some samples, additional images were collected but excluded from the analysis for consistency. Extra files are available at Caltech DATA (https://doi.org/10.22002/zg1d3-k3b49).
Replication	Large-scale 3D imaging was performed twice (Fig. 3 and Fig. 4). In both instances, large surfaces aggregates containing bacteria were detected. In the first pilot experiment, one biological replicate per group was imaged. In the second experiment, 4 biological replicates per group were imaged.
Randomization	Upon arrival, 21-day-old (just weaned) mice were randomly assigned to different cages/conditions by the veterinary staff (not the scientists conducting the experiment).
Blinding	Blinding was not possible because the same experimenter performed bacterial preparation for gavage, gavage, and sample collection.

Reporting for specific materials, systems and methods

We require information from authors about some types of materials, experimental systems and methods used in many studies. Here, indicate whether each material, system or method listed is relevant to your study. If you are not sure if a list item applies to your research, read the appropriate section before selecting a response.

Materials & experimental systems

n/a	Involved in the study	
☐	☒	Antibodies
☒	☐	Eukaryotic cell lines
☒	☐	Palaeontology and archaeology
☐	☒	Animals and other organisms
☒	☐	Human research participants
☒	☐	Clinical data
☒	☐	Dual use research of concern

Methods

n/a	Involved in the study	
☒	☐	ChIP-seq
☒	☐	Flow cytometry
☒	☐	MRI-based neuroimaging

Antibodies

Antibodies used	anti-EpCAM antibody conjugated to Alexafluor546 (sc-53532 AF546, lot C0519; Santa Cruz Biotechnology, Santa Cruz, CA, USA), anti-CD45 antibody conjugated to Alexafluor546 (sc-53665 AF546; lot C2619; Santa Cruz Biotechnology)
Validation	Vendor product sheets provide antibody validation data on samples of mouse origin. Furthermore, both antibodies were validated in house on mouse small intestine hydrogel-tissue hybrids. Anti-CD45 and anti-EpCAM antibodies stained immune cells in the core of the villi and enterocytes on the surface of the villi, respectively (Fig. S26).

Animals and other organisms

Policy information about studies involving animals; ARRIVE guidelines recommended for reporting animal research

Laboratory animals	C57BL/6J male mice were placed on experimental diets at 21-days-of-age and examined 28-31 days later at 49-52 days of age.
Wild animals	The study did not involve wild animals

Field-collected samples	The study did not involve samples collected from the field
Ethics oversight	All animal husbandry and experiments were approved by the Caltech Institutional Animal Care and Use Committee (IACUC, protocol #1646).

Note that full information on the approval of the study protocol must also be provided in the manuscript.
