# Supplementary material for: Quantitative whole-tissue 3D imaging reveals bacteria in close association with mouse jejunum mucosa
Source: NPJ Biofilms Microbiomes. 2023 Sep 7;9:64. doi: 10.1038/s41522-023-00423-2 (PMC10485000; doi:10.1038/s41522-023-00423-2)
Supplement: Supplementary file 2 — Supplementary Information [file 41522_2023_423_MOESM2_ESM.pdf]

Supplementary information for

Quantitative whole-tissue 3D imaging reveals bacteria in close association with mouse jejunum mucosa

Roberta Poceviciute,[1] Said R. Bogatyrev,[2,α] Anna E. Romano,[1] Amanda H. Dilmore,[2,β] Octavio Mondragón-Palomino,[1,γ] Heli Takko,[1,δ] Ojas Pradhan,[1] and Rustem F. Ismagilov[1,2*]

[1] Division of Chemistry & Chemical Engineering
[2] Division of Biology & Biological Engineering
California Institute of Technology 1200 E. California Blvd., Pasadena, CA, 91125 United States
* Correspondence to: rustem.admin@caltech.edu

[α] Current address: Medically Associated Science and Technology Program, Cedars-Sinai Medical Center, Los Angeles, CA, United States of America

[β] Current address: Biomedical Sciences Program, University of California San Diego, San Diego, CA, United States of America

[γ] Current address: Laboratory of Parasitic Diseases, National Institute of Allergy and Infectious Diseases, Bethesda, MD, United States of America

[δ] Current address: Department of Physics, University of Helsinki, University of Helsinki, Finland

Contents

- Supplementary Figures 1–26
- Supplementary Tables 1–2
- Supplementary Video and Data Captions
- Supplementary Methods
  - Validation of the universal degenerate HCR v3.0 probe set
  - Validation of the taxon-specific HCR v3.0 probes
  - Comparison of the taxon-specific HCR v2.0 and HCR v3.0 probe sets
  - Comparison of A1B.01P4 and A4B0P4 tissue gel formulations
  - Histopathology
- Supplementary References
- Detailed Author Contribution Statements

Supplementary Figures

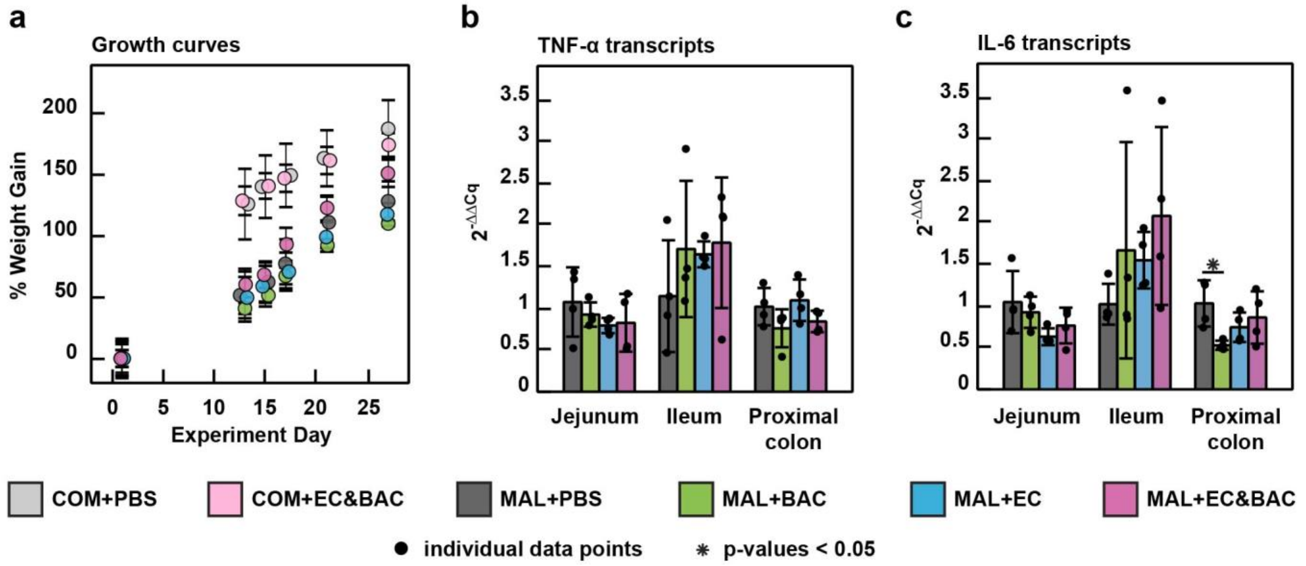

Supplementary Figure 1. Mouse response to dietary and bacterial interventions. (a) Mean weight gain data showing how malnutrition and bacterial gavage affected animal growth. Whiskers are ±S.D. (b–c) Relative quantification of TNF-α (b) and IL-6 (c) transcripts normalized to GAPDH housekeeping gene and with respect to the MAL+PBS group[1] using a TaqMan assay. Transcripts were quantified in the jejunum (2nd quartile of the SI), ileum (4th quartile of the SI), and proximal colon tissues in malnourished mice only. Each group contained four mice, which were euthanized over the course of 4 days, one mouse per group per day. Individual biological replicates (each of which is the average of triplicate qPCR runs) are plotted as dots and group averages are plotted as bars. Whiskers are ±S.D. Kruskal–Wallis test was used to evaluate statistical significance between treatments within each tissue type, and Benjamini–Hochberg correction was used to account for multiple comparisons. COM: nutritionally complete diet. MAL: malnourishing diet low in protein (7%) and fat (5%), but containing the same amounts of calories, vitamins, and minerals on the basis of mass. PBS: phosphate buffered saline gavage as a no-bacteria control. BAC: gavage with five *Bacteroides*/*Parabacteroides* spp. isolates. EC: gavage with two *E. coli* isolates. EC&BAC: gavage with all seven bacterial isolates.

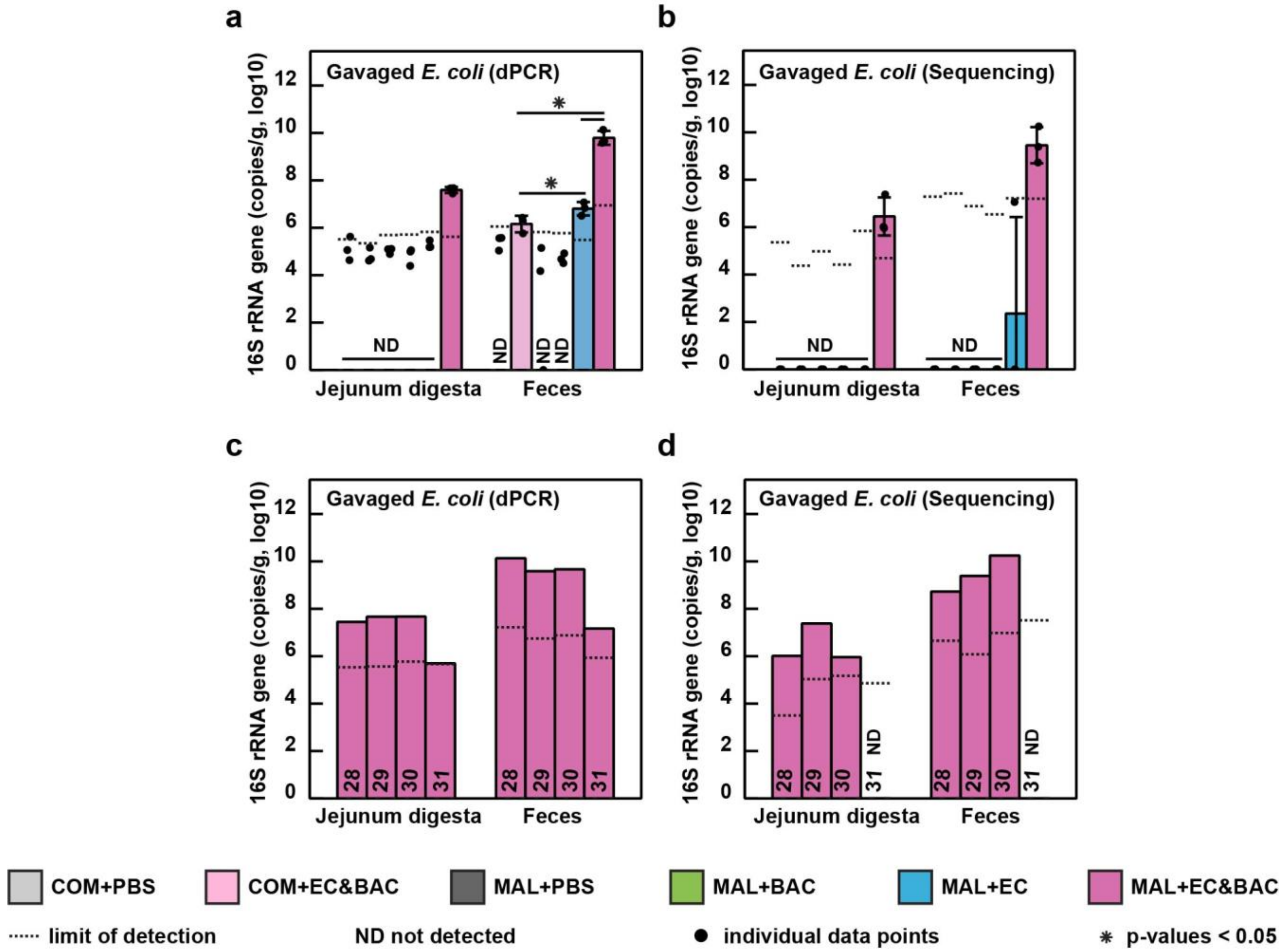

Supplementary Figure 2. Absolute abundance of *E. coli* 16S rRNA gene copies in jejunum digesta and feces across treatment groups. (a) Log-absolute 16S rRNA gene copy abundance of gavaged *E. coli* quantified by digital PCR (dPCR) with *Enterobacteriaceae* primers. (b) Extrapolation of log-absolute 16S rRNA gene copy abundance of gavaged *E. coli* using 16S rRNA gene amplicon sequencing data (Fig. 2a) and total 16S rRNA gene copy quantification by dPCR with universal bacterial primers (Fig. 2b)[2]. Panel b is identical to Fig. 2d and is displayed here for comparison purposes. In panels a and b, the analysis consisted of six groups: control mice not gavaged with bacteria (COM+PBS), control mice gavaged with an *E. coli* and *Bacteroides*/*Parabacteroides* spp. cocktail (COM+EC&BAC), malnourished mice not gavaged with bacteria (MAL+PBS), malnourished mice gavaged only with *Bacteroides*/*Parabacteroides* spp. isolates (MAL+BAC), malnourished mice gavaged only with *E. coli* isolates (MAL+EC), and malnourished mice gavaged with the full bacterial cocktail (MAL+EC&BAC). Each group contained three mice, which were euthanized on days 28, 29, and 30, with one mouse per group per day. The dots show individual biological replicates and bars show group averages. Whiskers are ±S.D. Limit of detection is expressed as group average. Statistical significance was evaluated using Kruskal-Wallis tests. (c) Log-absolute 16S rRNA gene copy abundance of gavaged *E. coli* quantified by dPCR with *Enterobacteriaceae* primers in MAL+EC&BAC group. (d) Extrapolation of log-absolute 16S rRNA gene copy abundance of gavaged *E. coli* in MAL+EC&BAC group using 16S rRNA gene amplicon sequencing data (Fig. 2a) and total 16S rRNA gene copy quantification by dPCR with universal bacterial primers (Fig. 2b). In c and d, each bar represents an individual mouse euthanized on the specified day of the experiment.

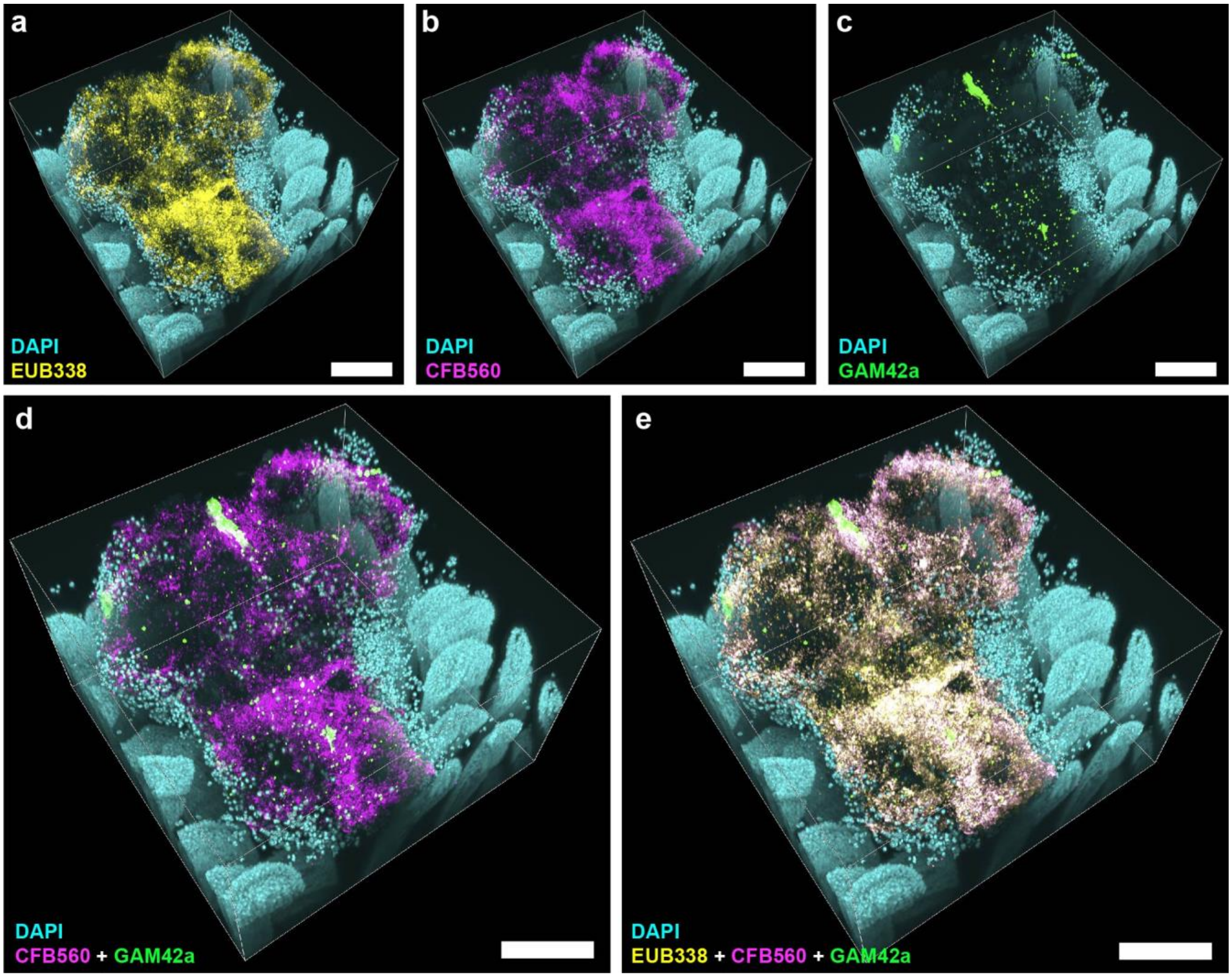

Supplementary Figure 3. High-magnification 3D imaging of a large surface aggregate detected in a MAL+EC&BAC mouse. (a-e) High-magnification 3D fluorescence image of a large surface aggregate seen in Fig. 3b (red square) showing DAPI staining of epithelium (cyan) and HCR v2.0 staining of (a) total bacteria with EUB338 probe (yellow), (b) *Bacteroidetes* with CFB560 probe (magenta), (c) *Gammaproteobacteria* with GAM42a probe (green), (d) *Bacteroidetes* and *Gammaproteobacteria*, and (e) total bacteria, *Bacteroidetes*, and *Gammaproteobacteria*. Supplementary Fig. 3 shows the same large surface aggregate as shown in Fig. 3c but is displayed here at an enlarged scale. All scale bars 200 µm.

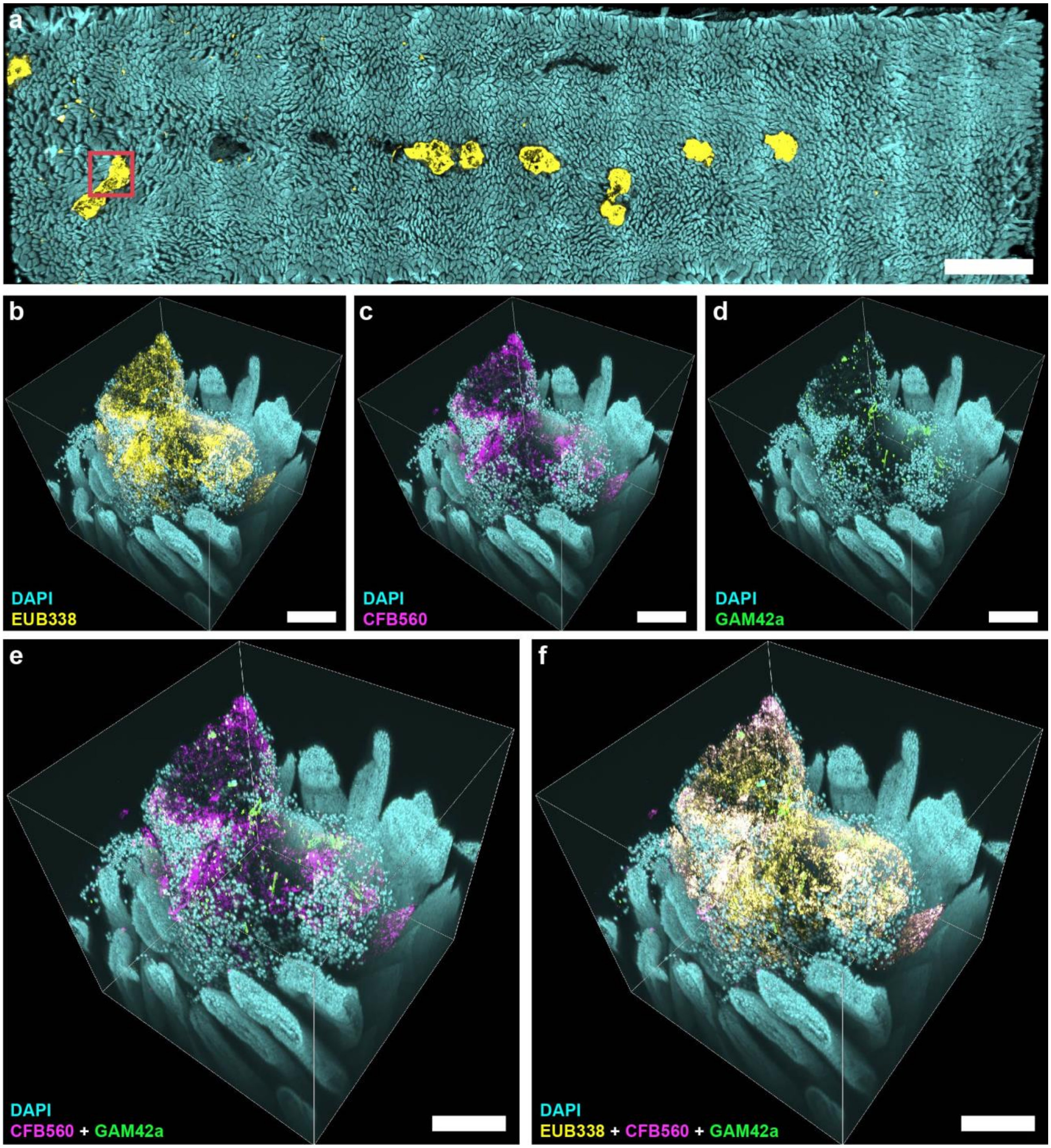

Supplementary Figure 4. High-magnification 3D imaging of a large surface aggregate detected in a MAL+EC&BAC mouse. (a) Large-scale low-magnification fluorescence tile scan of a whole empty jejunum segment showing DAPI staining of epithelial surface (cyan) and HCR v2.0 staining of total bacteria with EUB338 probe (yellow). Red square marks the position where high magnification 3D image shown in panels b-f was acquired. Scale bar 2 mm. (b-f) High-magnification 3D fluorescence image of a large surface aggregate showing DAPI staining of epithelium (cyan) and HCR v2.0 staining of (b) total bacteria with EUB338 probe (yellow), (c) *Bacteroidetes* with CFB560 probe (magenta), (d) *Gammaproteobacteria* with GAM42a probe (green), (e) *Bacteroidetes* and *Gammaproteobacteria*, and (f) total bacteria, *Bacteroidetes*, and *Gammaproteobacteria*. This is the second of two large surface aggregates imaged at higher magnification; the other is shown in Fig. 3c and Supplementary Fig. 3. All scale bars 200 µm.

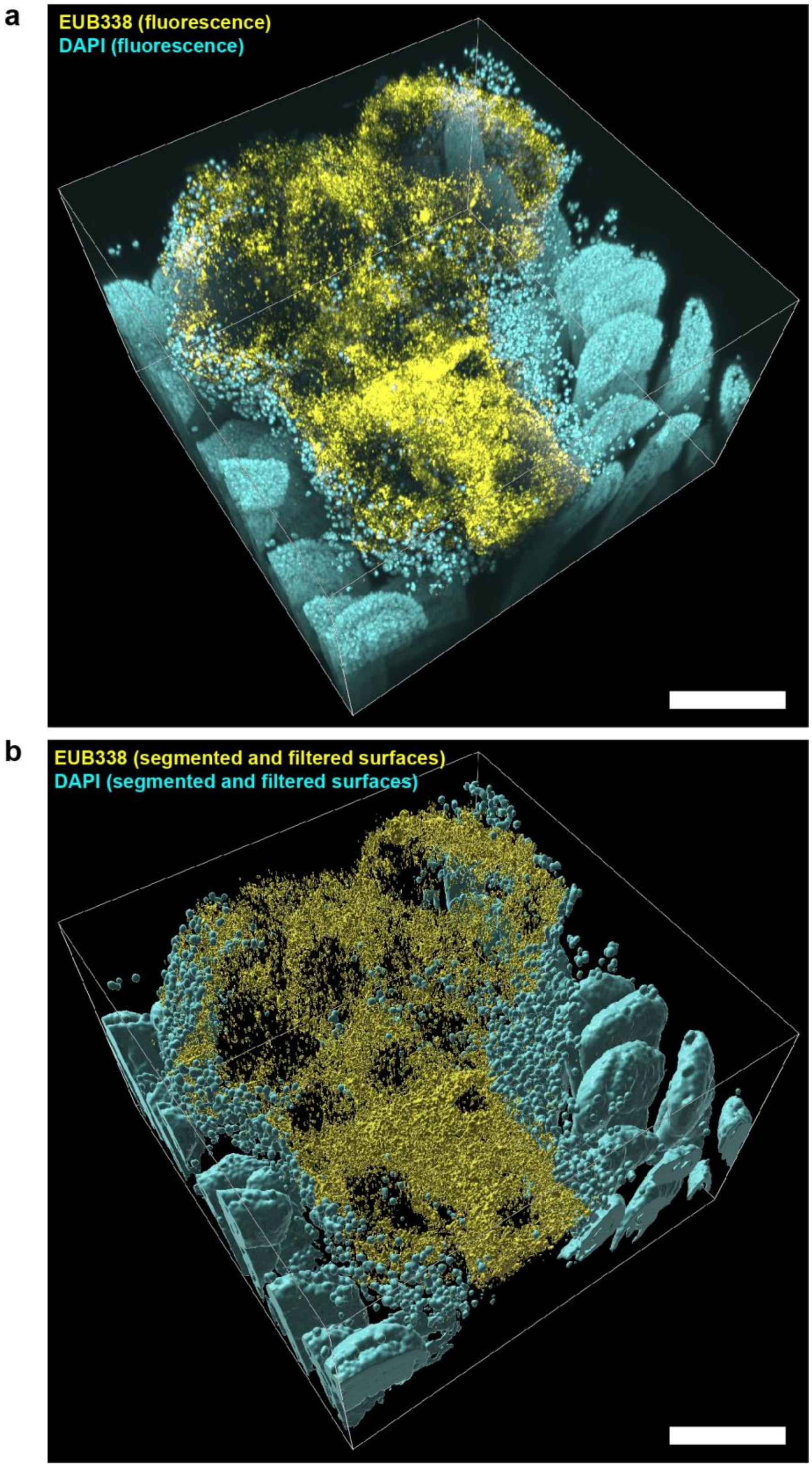

Supplementary Figure 5. Example image segmentation and filtering of total bacteria and host epithelium for a large surface aggregate detected in a MAL+EC&BAC mouse. (a) High-magnification 3D fluorescence image before segmentation and filtering of a large surface aggregate seen in Fig. 3b (red square) showing DAPI staining of epithelium (cyan) and HCR v2.0 staining of total bacteria with EUB338 probe (yellow). (b) 3D rendering of segmented and filtered image showing DAPI+ and EUB338+ surfaces. All scale bars 200 µm.

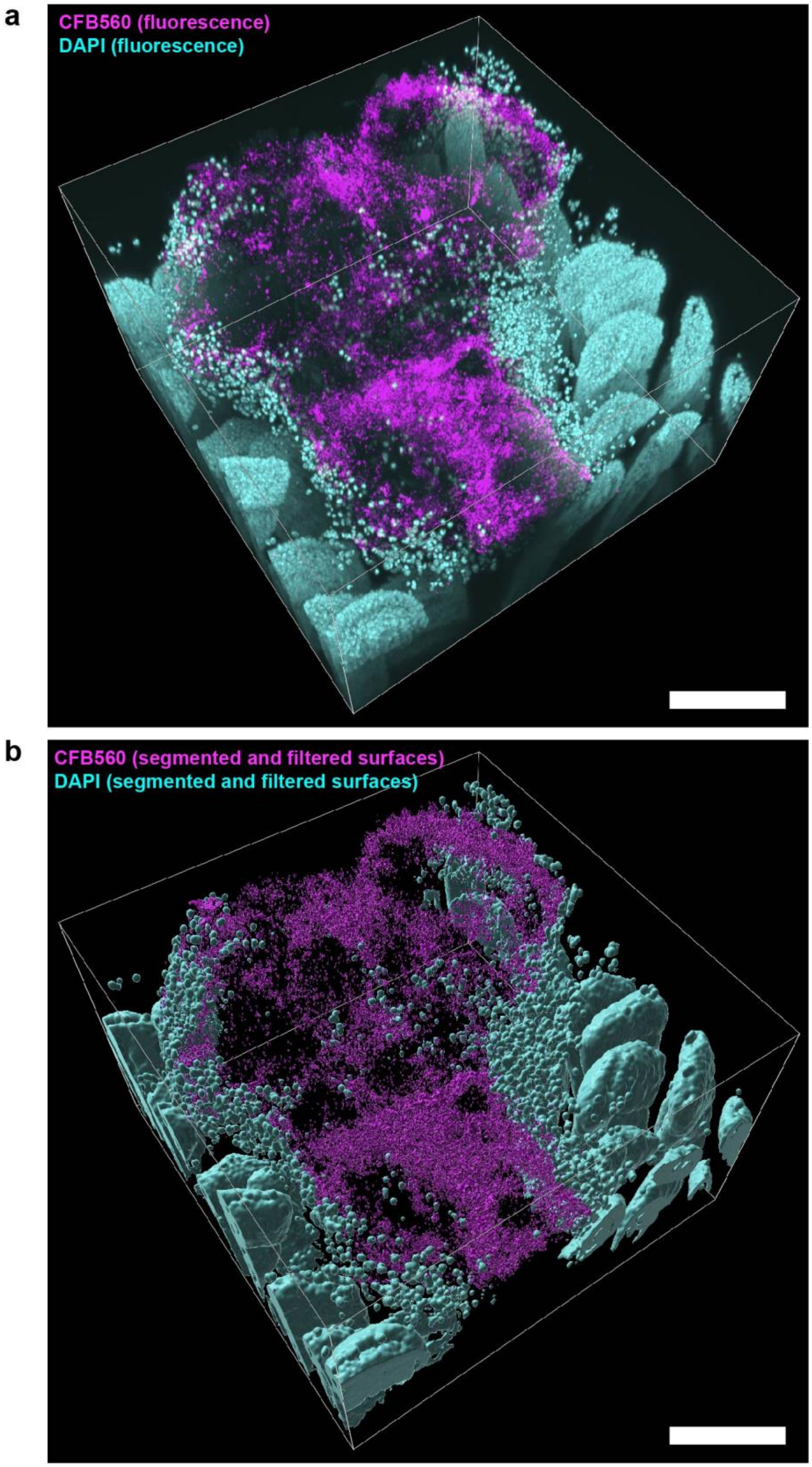

Supplementary Figure 6. Example image segmentation and filtering of *Bacteroidetes* and host epithelium for a large surface aggregate detected in a MAL+EC&BAC mouse. (a) High-magnification 3D fluorescence image before segmentation and filtering of a large surface aggregate seen in Fig. 3b (red square) showing DAPI staining of epithelium (cyan) and HCR v2.0 staining of *Bacteroidetes* with CFB560 probe (magenta). (b) 3D rendering of segmented and filtered image showing DAPI+ and CFB560+ surfaces. All scale bars 200 µm.

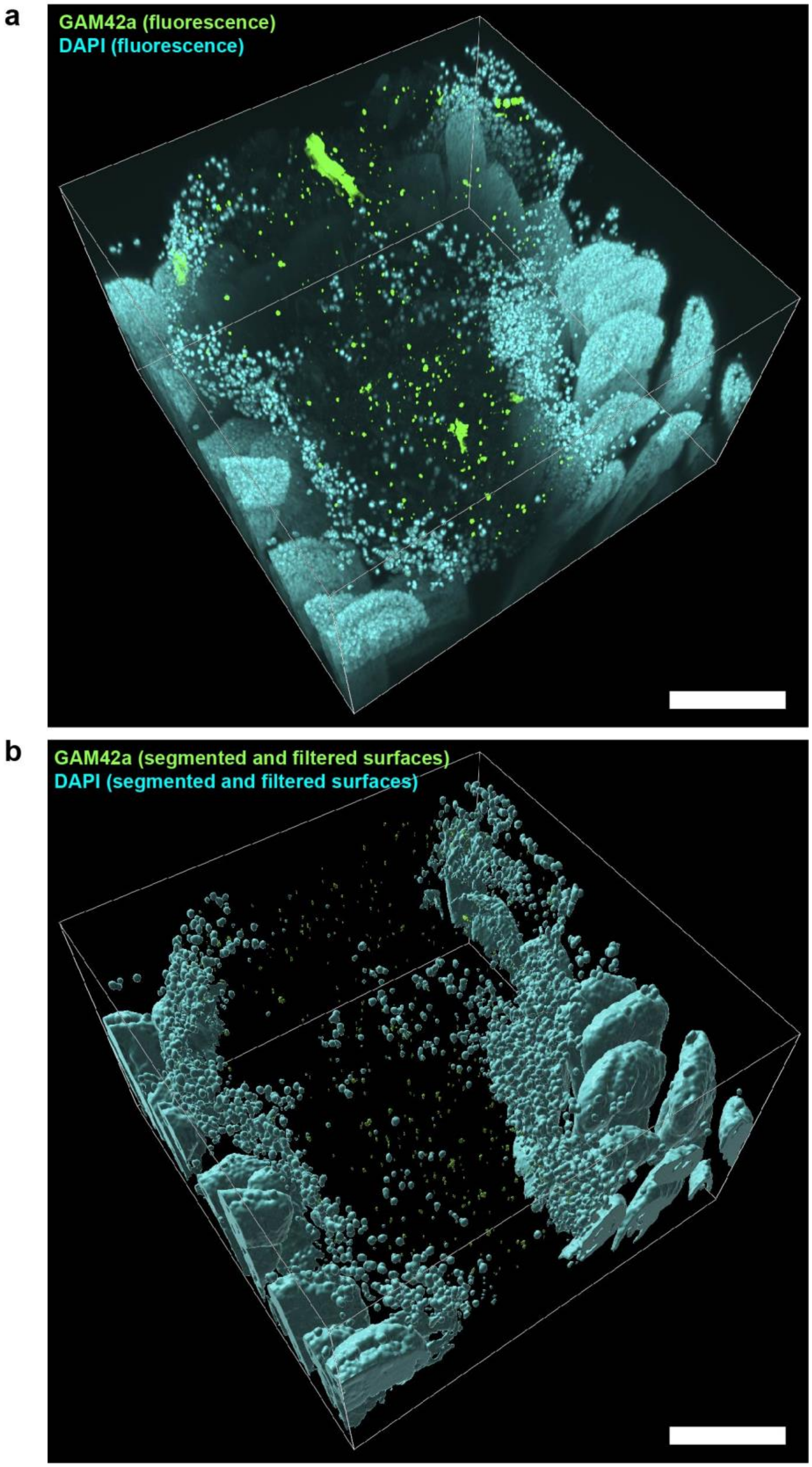

Supplementary Figure 7. Example image segmentation and filtering of *Gammaproteobacteria* and host epithelium for a large surface aggregate detected in a MAL+EC&BAC mouse. (a) High-magnification 3D fluorescence image before segmentation and filtering of a large surface aggregate seen in Fig. 3b (red square) showing DAPI staining of epithelium (cyan) and HCR v2.0 staining of *Gammaproteobacteria* with GAM42a probe (magenta). (b) 3D rendering of segmented and filtered image showing DAPI+ and GAM42a+ surfaces. Large objects in GAM42a channel did not overlap with EUB338 total bacterial staining and were removed during filtering as false positives. All scale bars 200 µm.

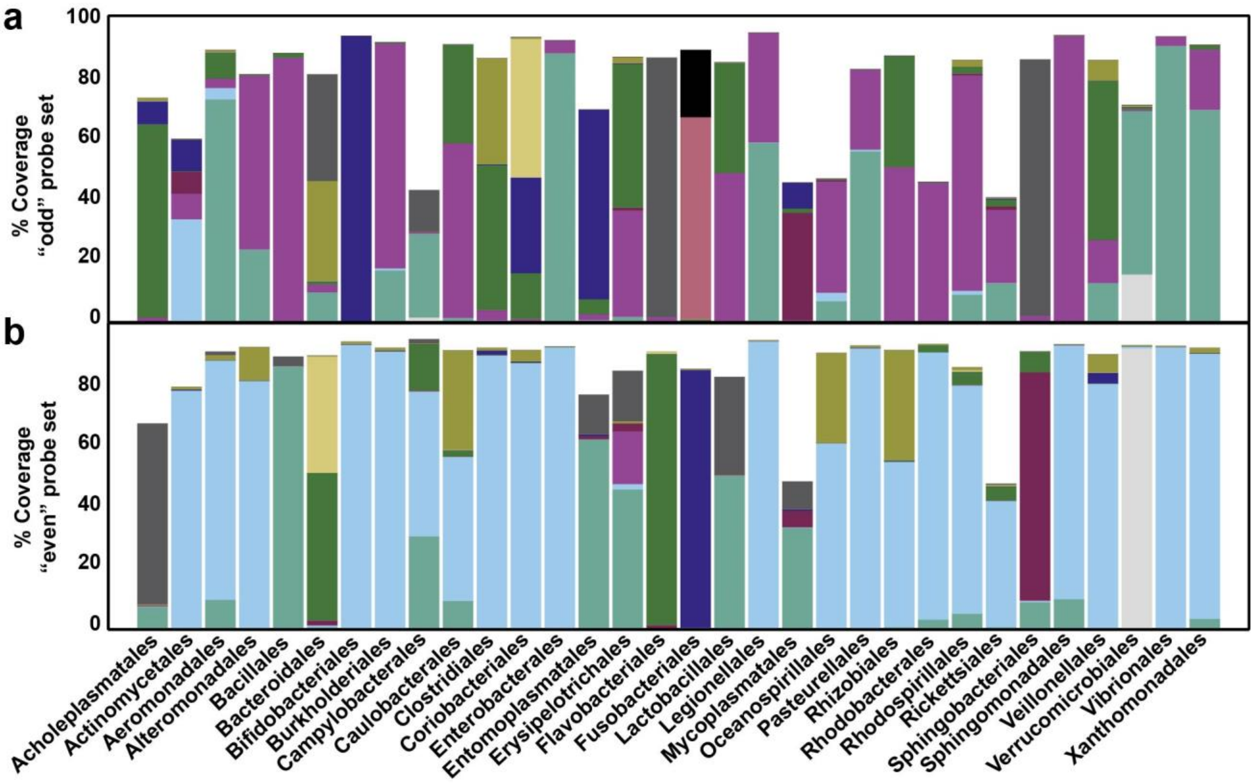

Supplementary Figure 8. Design of the universal degenerate HCR v3.0 probe set. HCR v3.0 achieves suppression of off-target signal amplification by requiring two target-bound probes to initiate signal amplification, which decreases the likelihood that the non-specifically bound probes are amplified[3]. To design HCR v3.0 probes for total bacteria, a 52-bp region that overlaps with the EUB338 binding site was first selected by Molecular Technologies. To improve coverage, we expanded this region to 17 full degenerate probes, and analyzed the coverage of 10 and 12 split probes split in the center, referred here as “odd” (a) and “even” (b). The location of the split is proprietary (and it actually yields 6 and 12 probes); therefore, we analyzed the coverage of the hypothetical 26-bp-long split probes. The height of each colored bar represents the percentage of coverage without a mismatch by one degenerate split probe, and the net bar height represents cumulative coverage by the degenerate probe set of the bacterial order of interest. Coverage is defined as % of order-specific 16S rRNA sequences in the SILVA 138 NR99 database that align with the probe without a mismatch. Only common bacterial orders were considered in the analysis.

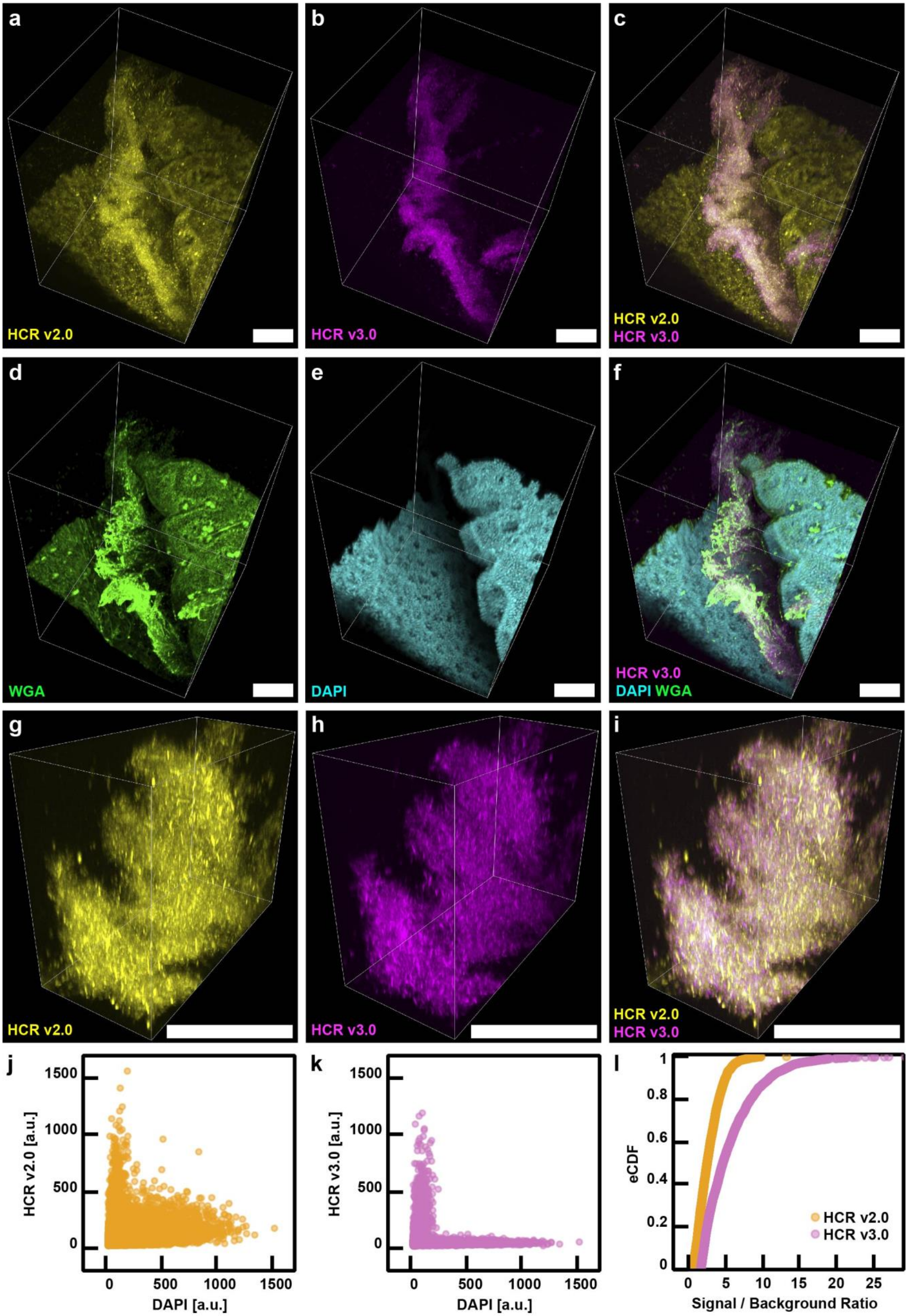

a
HCR v2.0
b
HCR v3.0
c
HCR v2.0
HCR v3.0
d
WGA
e
DAPI
f
HCR v3.0
DAPI WGA
g
HCR v2.0
h
HCR v3.0
i
HCR v2.0
HCR v3.0
j
HCR v2.0 [a.u.]
DAPI [a.u.]
k
HCR v3.0 [a.u.]
DAPI [a.u.]
l
eCDF
Signal / Background Ratio
HCR v2.0
HCR v3.0

Supplementary Figure 9. Comparison of the universal degenerate HCR v3.0 probe set and EUB338 HCR v2.0 probe in a proximal colon of CHOW-fed control mouse. (a-i) CLARITY imaging of a mouse proximal colon with the full acquired z-stack shown in a-f and the zoomed in portions focused on bacteria shown in g-i. All scale bars 100 µm. (a) Bacteria tagging with EUB338 HCR v2.0 probe (yellow). (b) Bacteria tagging with the universal degenerate HCR v3.0 probe set (magenta). (c) Overlay of panels a-b showing that the signals overlapped in the center of the image, i.e. in between two proximal colon folds. (d) Epithelium staining with DAPI (cyan) showing proximal colon folds and a void in between. (e) Mucus staining with WGA lectin (green) showing mucus-filled goblet cells in the tissue and secreted mucus in between proximal colon folds. (f) Overlay of b, d, and e showing spatial structure of tissue, secreted mucus and bacteria tagged by HCR v3.0. (j-k) Voxel intensity analysis of a sub-sampled voxel population in panels a-f. (j) HCR v2.0 signal vs DAPI signal, showing that voxels well-stained by DAPI also had elevated HCR v2.0 signal. (k) HCR v3.0 signal vs DAPI signal, showing that voxels well-stained by DAPI segregated from voxels well-stained by HCR v3.0. (l) Empirical cumulative distribution function (eCDF) of signal-to-background ratio of HCR v2.0 and HCR v3.0 staining in the zoomed-in image shown in g-i. eCDF plots show that, relative to HCR v2.0, HCR v3.0 increased signal-to-background ratio.

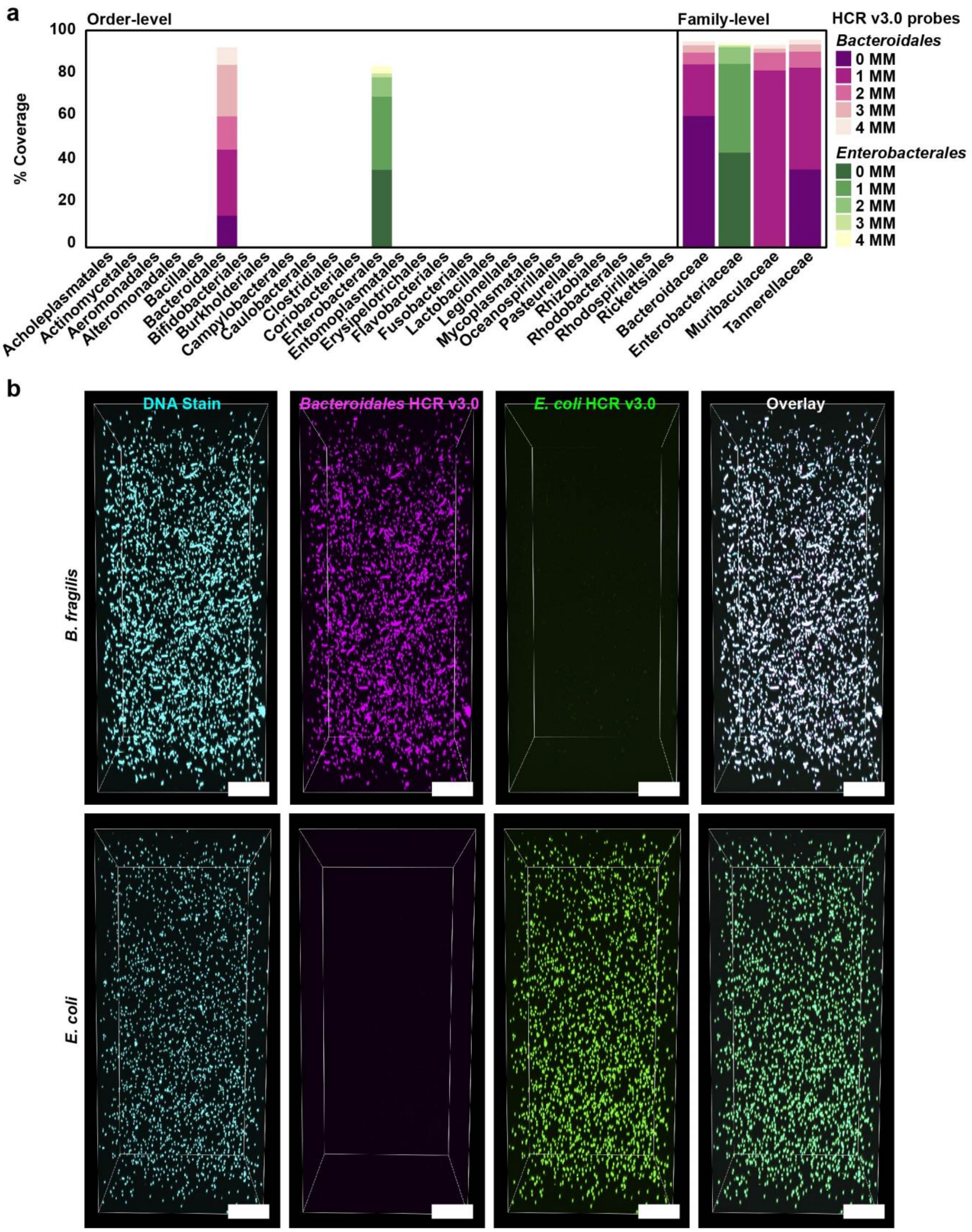

Supplementary Figure 10. Design and validation of the taxon-specific HCR v3.0 probes. HCR v3.0 probes specific to *Bacteroidales* and *E. coli* were designed by Molecular Technologies to have no mismatches with the full 16S rRNA gene sequences of the gavaged *Bacteroides*/*Parabacteroides* spp. and *E. coli* isolates, respectively. (a) Probe coverage with no mismatches (0 MM) or up to 4 mismatches (1-4 MM) of all bacterial orders (left) and relevant bacterial families (right) detected (by sequencing) in the experimental mice. The coverage is expressed as % of sequences in SILVA database that align with the full 52-bp long HCR v3.0 probes when the specified number of mismatches is allowed. (b) Probe validation *in vitro* showing that the probes recognized their targets but did not cross-react with model off-targets even deep in the hydrogel. The validation was performed using single-isolate *in vitro* hydrogels, either *E. coli* or *B. fragilis*. Both hydrogels were stained with the two taxon-specific HCR v3.0 probes (*Bacteroidales*: magenta; *E. coli*: green) and counterstained with a nuclear stain (cyan). All scale bars 100 µm.

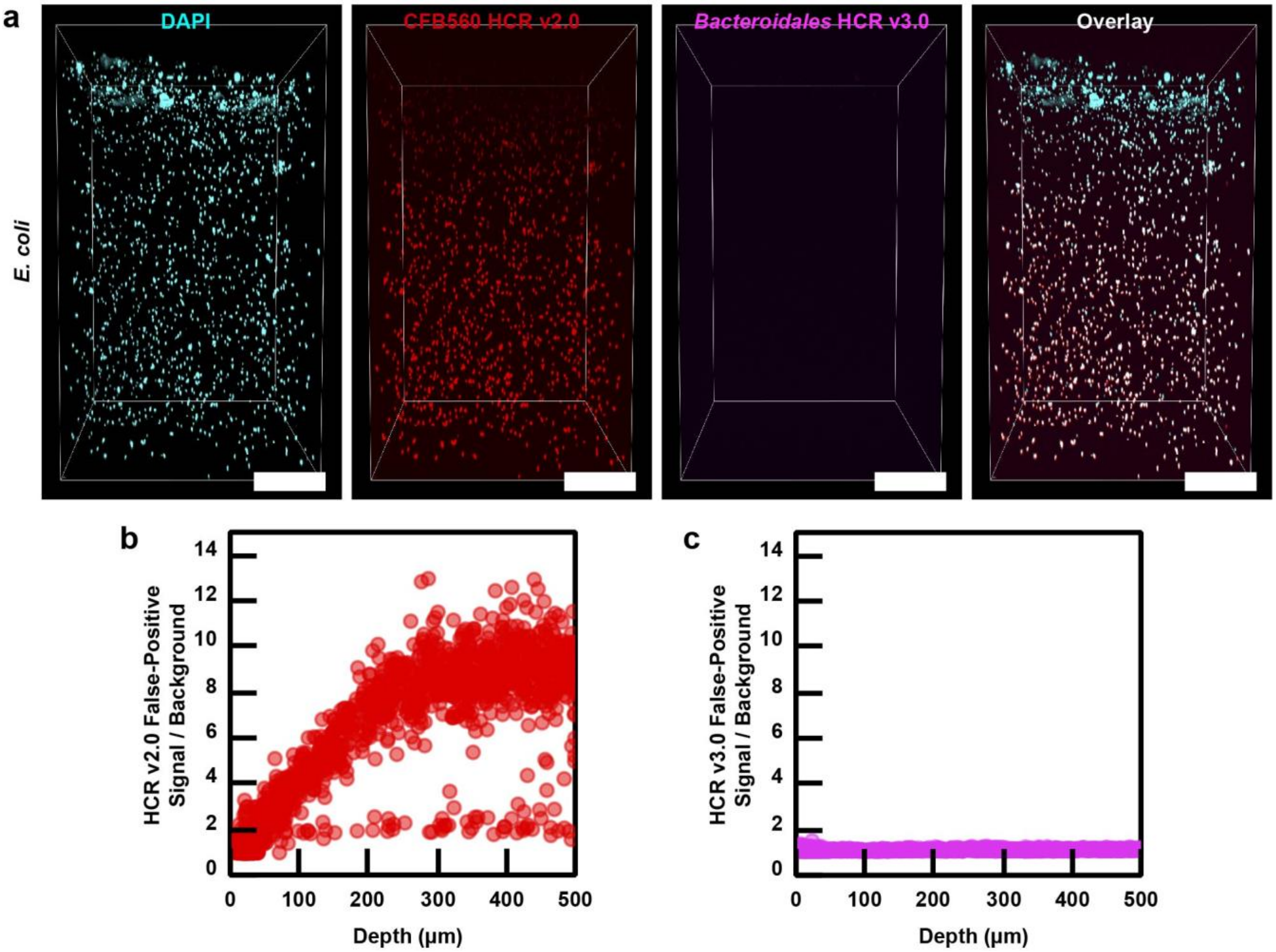

Supplementary Figure 11. Comparison of the taxon-specific *Bacteroidales* HCR v3.0 and CFB560 HCR v2.0 probes in an *in vitro* hydrogel with *E. coli*. (a) *E. coli* staining with DAPI for DNA (cyan), *Bacteroidetes*-specific CFB560 HCR v2.0 probe (red), and *Bacteroidales* HCR v3.0 probe (magenta). All scale bars 100 µm. (b-c) Signal-to-background ratio of (b) CFB560 v2.0 and (c) *Bacteroidales* HCR v3.0 probe staining of *E. coli* cells versus hydrogel depth. Each data point represents a single *E. coli* cell obtained by segmentation in the DAPI channel. The CFB560 HCR v2.0 probe suffered from increasing false-positive signal amplification with depth, whereas the *Bacteroidales* HCR v3.0 probe successfully suppressed false-positive signal amplification deep into the hydrogel.

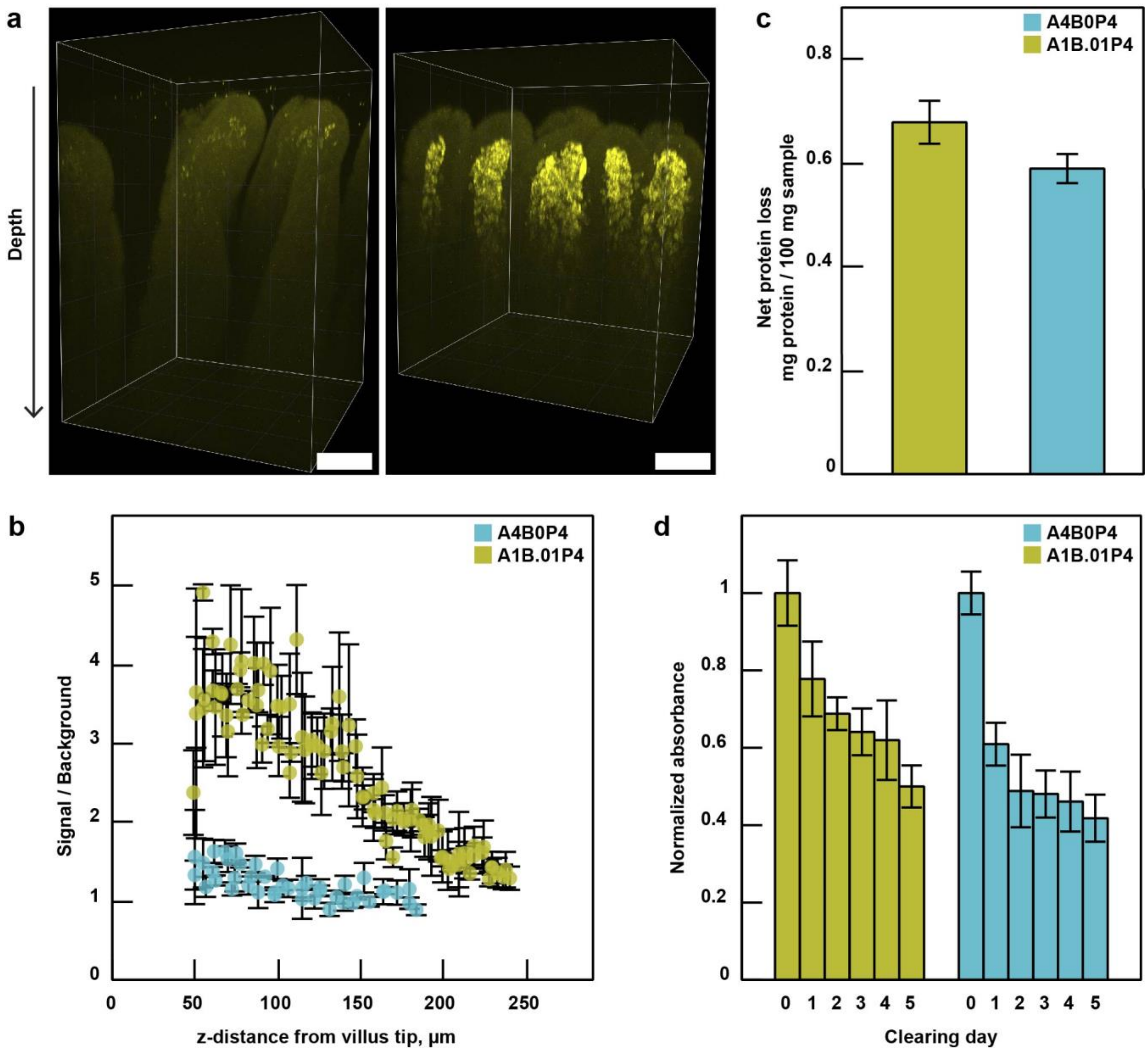

Supplementary Figure 12. Comparison of the more permeable A1B.01P4 and the less permeable A4B0P4 tissue gel formulations. (a) 3D imaging z-stacks of anti-CD45 antibody staining of total immune cells (yellow) in a small intestine of CHOW-fed control mouse. In both images, the mucosa was protected by the same surface hydrogel, but the tissues were additionally fortified with either a 4% acrylamide + 4% paraformaldehyde formulation (A4B0P4) (left) or a 1% acrylamide + 0.01% bis-acrylamide + 4% paraformaldehyde (A1B.01P4) hydrogel formulation (right). All scale bars 100 µm. (b) Quantification of antibody penetration into the hydrogel-tissue hybrids shown in panel a, demonstrating that A1B.01P4 chemistry increased the antibody signal/background ratio deep in the sample. (c) Net protein loss during clearing shows that a lower percentage of acrylamide hydrogel did not increase protein loss. (d) Normalized tissue absorbance during clearing shows that tissue clarity was similar for both hydrogel formulations.

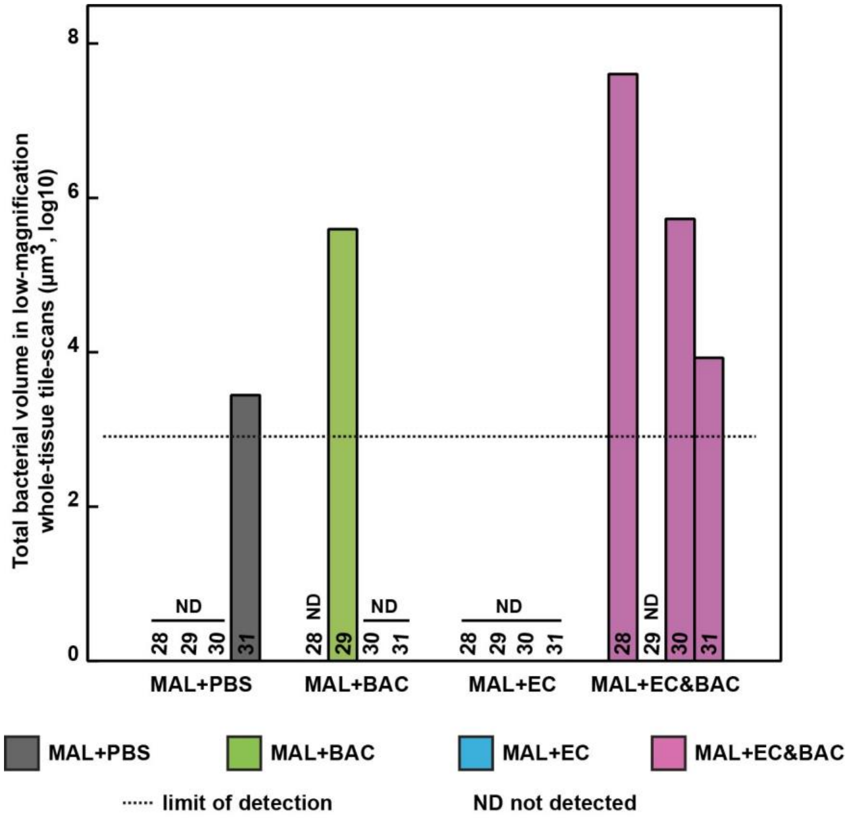

Supplementary Figure 13. Quantification of total bacterial volume in large-scale low-magnification tile scans of empty jejunum segments. Mice were euthanized on days 28-31, with one mouse per group per day. Total bacteria were stained by HCR v3.0. In all samples, total bacteria were tagged with Alexafluor594 fluorophore; the only exception was MAL+EC&BAC day 30 sample, in which total bacteria were tagged with Alexfluor514 fluorophore in a preliminary staining experiment. Segmentation and filtering settings for quantification of total bacteria are provided in Supplementary Data 4. Example images are shown in Fig. 4b and Supplementary Figs. 14-19. MAL: malnourishing diet. PBS: phosphate buffered saline gavage as a no-bacteria control. BAC: gavage with five *Bacteroides*/*Parabacteroides* spp. isolates. EC: gavage with two *E. coli* isolates. EC&BAC: gavage with all seven bacterial isolates. Bacteria detected in MAL+PBS mouse euthanized on day 31 was orders of magnitude lower than in mice with large surface aggregates and was not apparent by visual inspection (Supplementary Fig. 14).

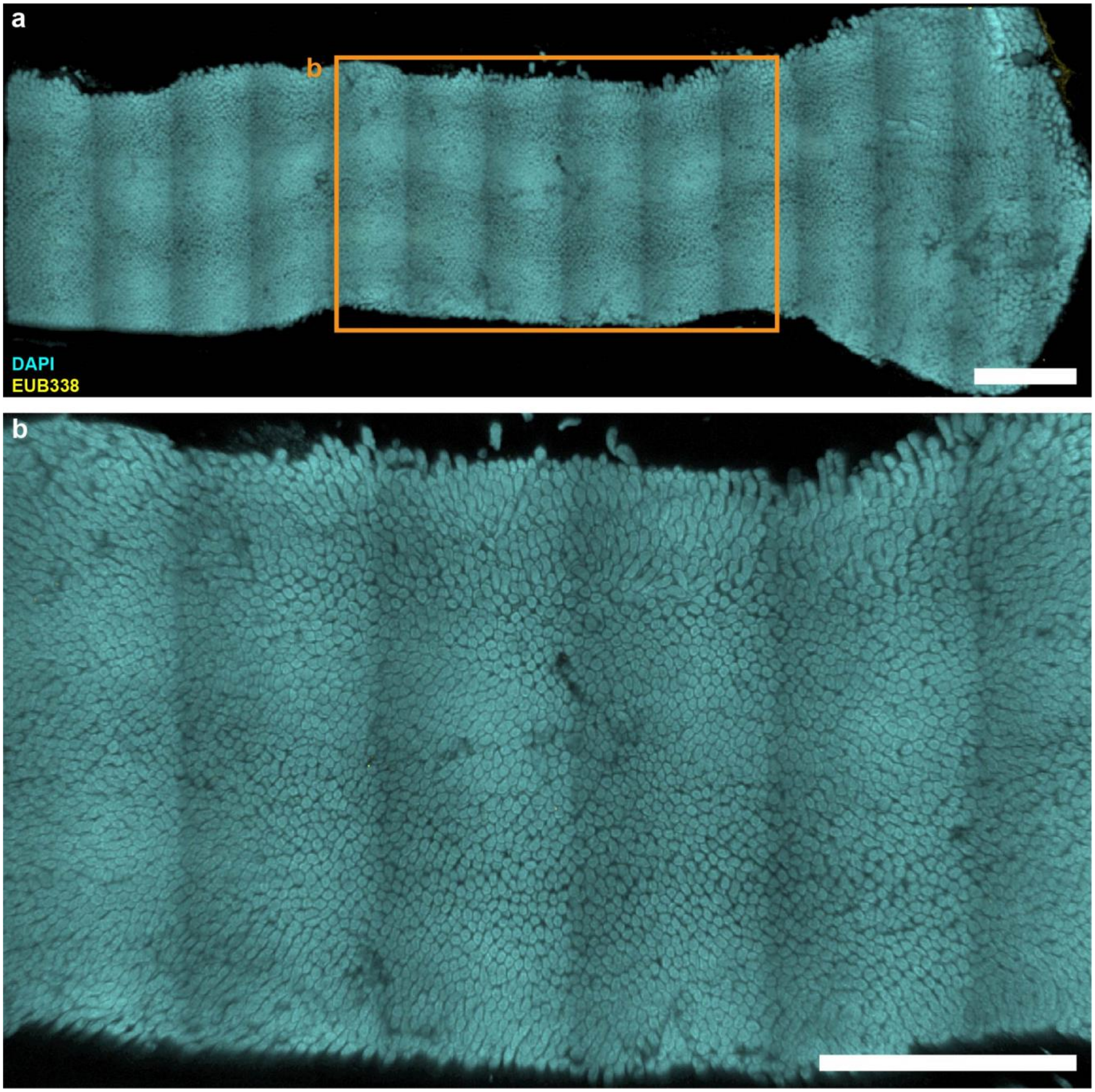

Supplementary Figure 14. Large-scale low-magnification fluorescence imaging of empty jejunum from MAL+PBS mouse on day 31 of the experiment. (a) Large-scale low-magnification tile scan of whole empty jejunum segment without digesta showing DAPI staining of epithelial surface (cyan) and HCR v3.0 staining of total bacteria (yellow). (b) Enlarged portion of the image shown in a (orange rectangle). All scale bars 2 mm.

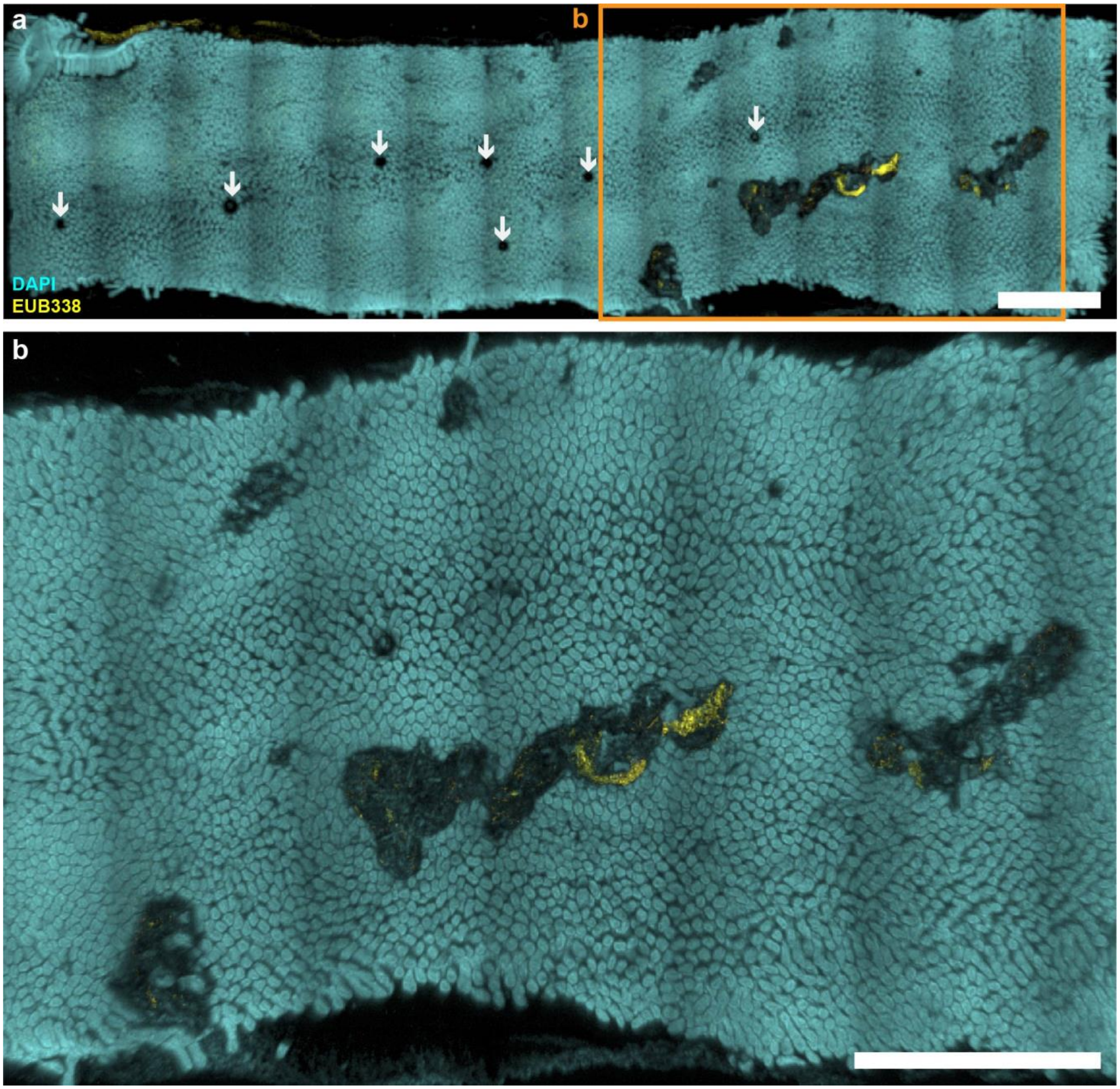

Supplementary Figure 15. Large-scale low-magnification fluorescence imaging of empty jejunum from MAL+BAC mouse on day 29 of the experiment. (a) Large-scale low-magnification tile scan of whole empty jejunum segment without digesta showing DAPI staining of epithelial surface (cyan) and HCR v3.0 staining of total bacteria (yellow). Small circles (white arrows) are imaging artifacts created by air bubbles trapped under the cover slip. (b) Enlarged portion of the image shown in a (orange rectangle). All scale bars 2 mm.

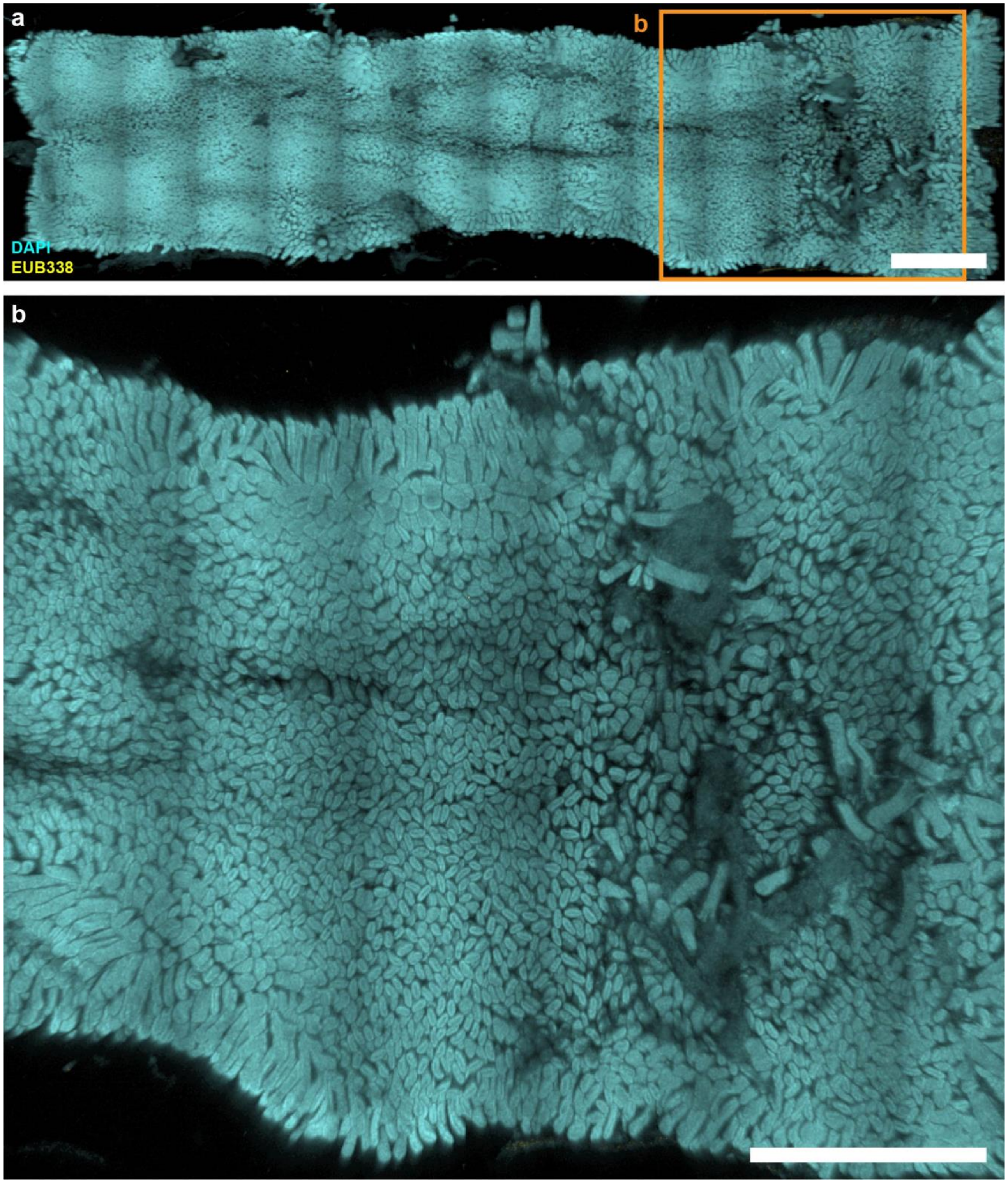

Supplementary Figure 16. Large-scale low-magnification fluorescence imaging of empty jejunum from MAL+EC mouse on day 28 of the experiment. (a) Large-scale low-magnification tile scan of whole empty jejunum segment without digesta showing DAPI staining of epithelial surface (cyan) and HCR v3.0 staining of total bacteria (yellow). (b) Enlarged portion of the image shown in a (orange rectangle). In this part of the sample, villus loss is clearly visible. All scale bars 2 mm.

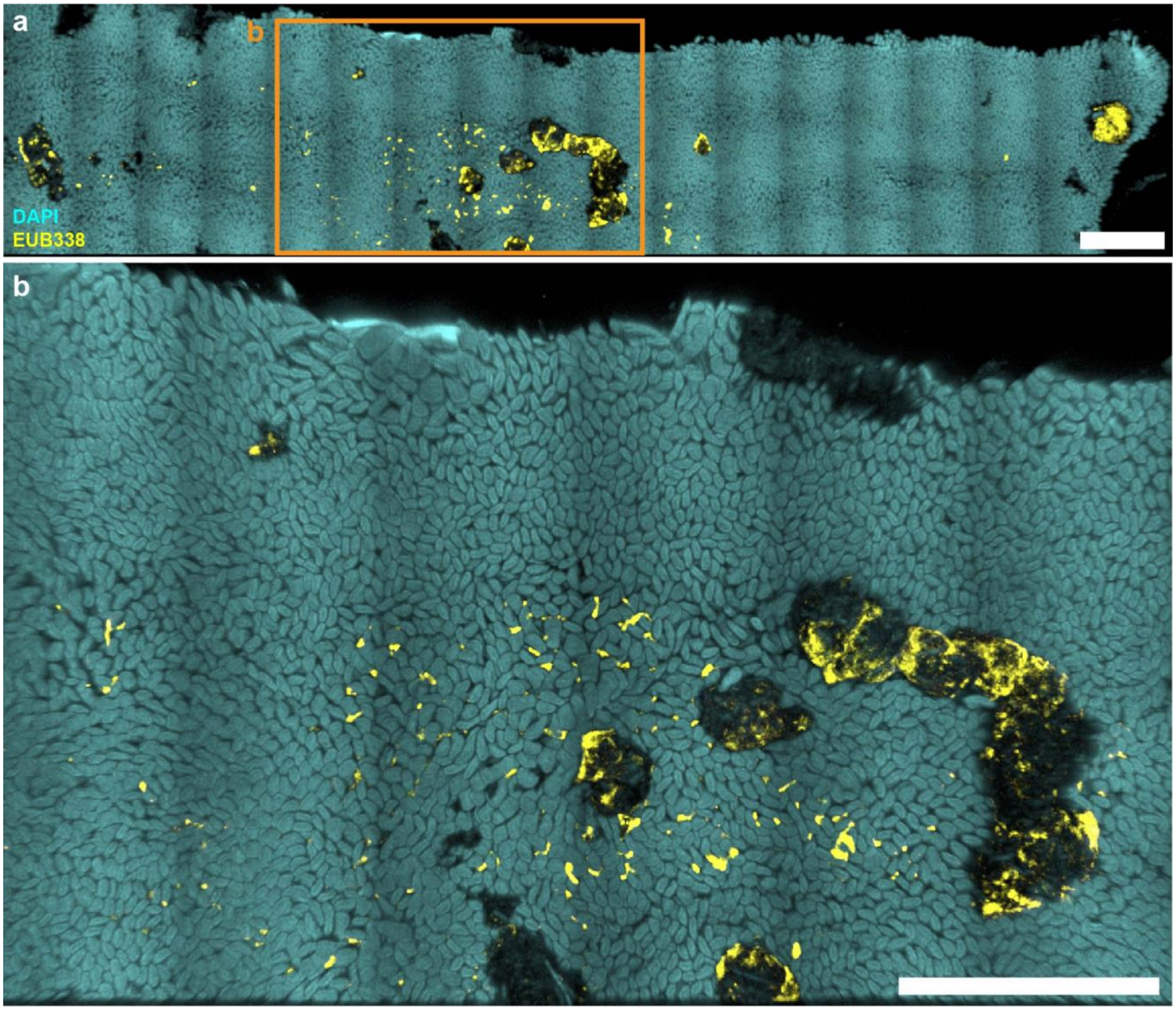

Supplementary Figure 17. Large-scale low-magnification fluorescence imaging of empty jejunum from MAL+EC&BAC mouse on day 28 of the experiment. (a) Large-scale low-magnification tile scan of whole empty jejunum segment without digesta showing DAPI staining of epithelial surface (cyan) and HCR v3.0 staining of total bacteria (yellow). (b) Enlarged portion of the image shown in a (orange rectangle). All scale bars 2 mm.

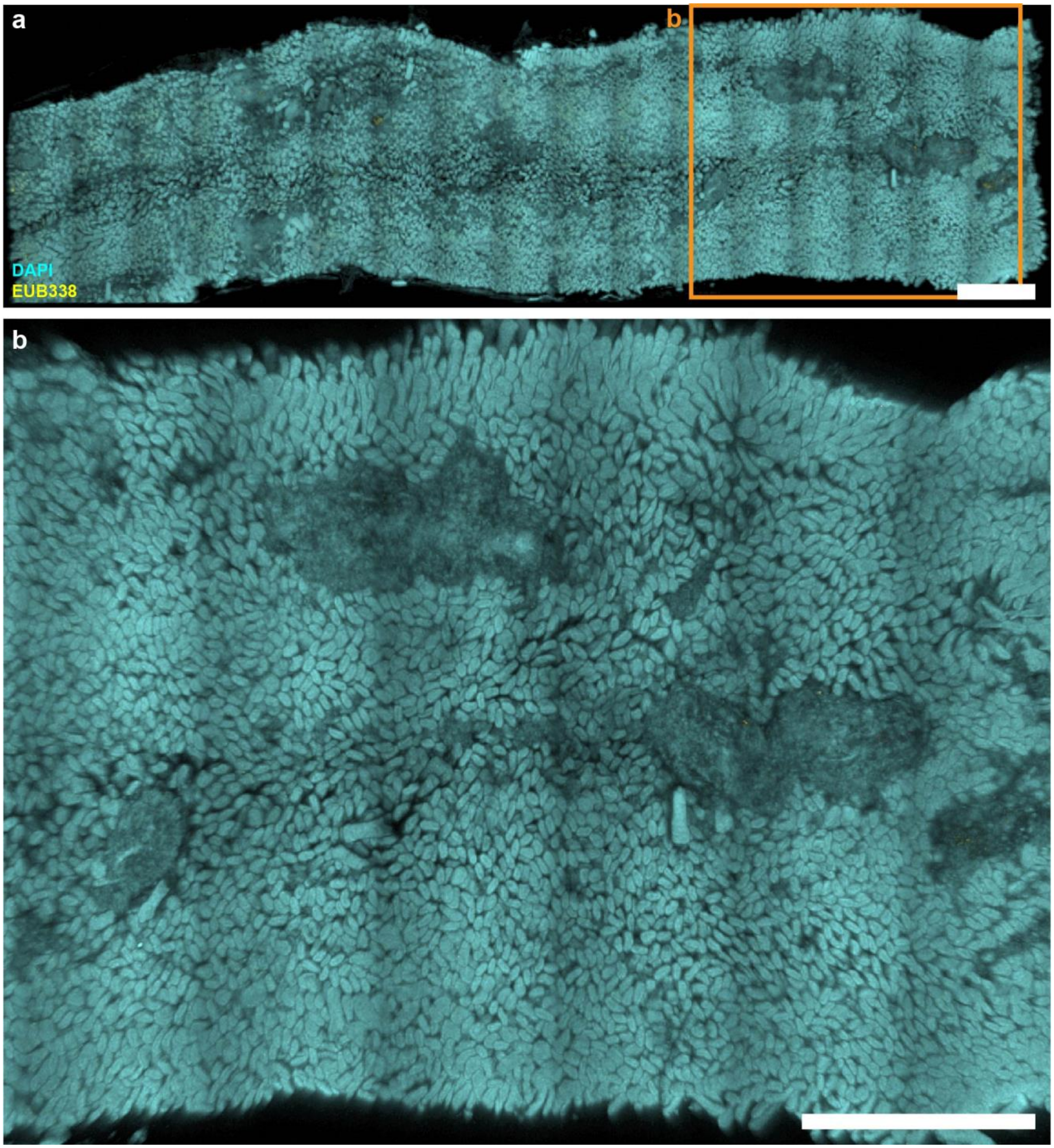

Supplementary Figure 18. Large-scale low-magnification fluorescence imaging of empty jejunum from MAL+EC&BAC mouse on day 31 of the experiment. (a) Large-scale low-magnification tile scan of whole empty jejunum segment without digesta showing DAPI staining of epithelial surface (cyan) and HCR v3.0 staining of total bacteria (yellow). (b) Enlarged portion of the image shown in a (orange rectangle). All scale bars 2 mm.

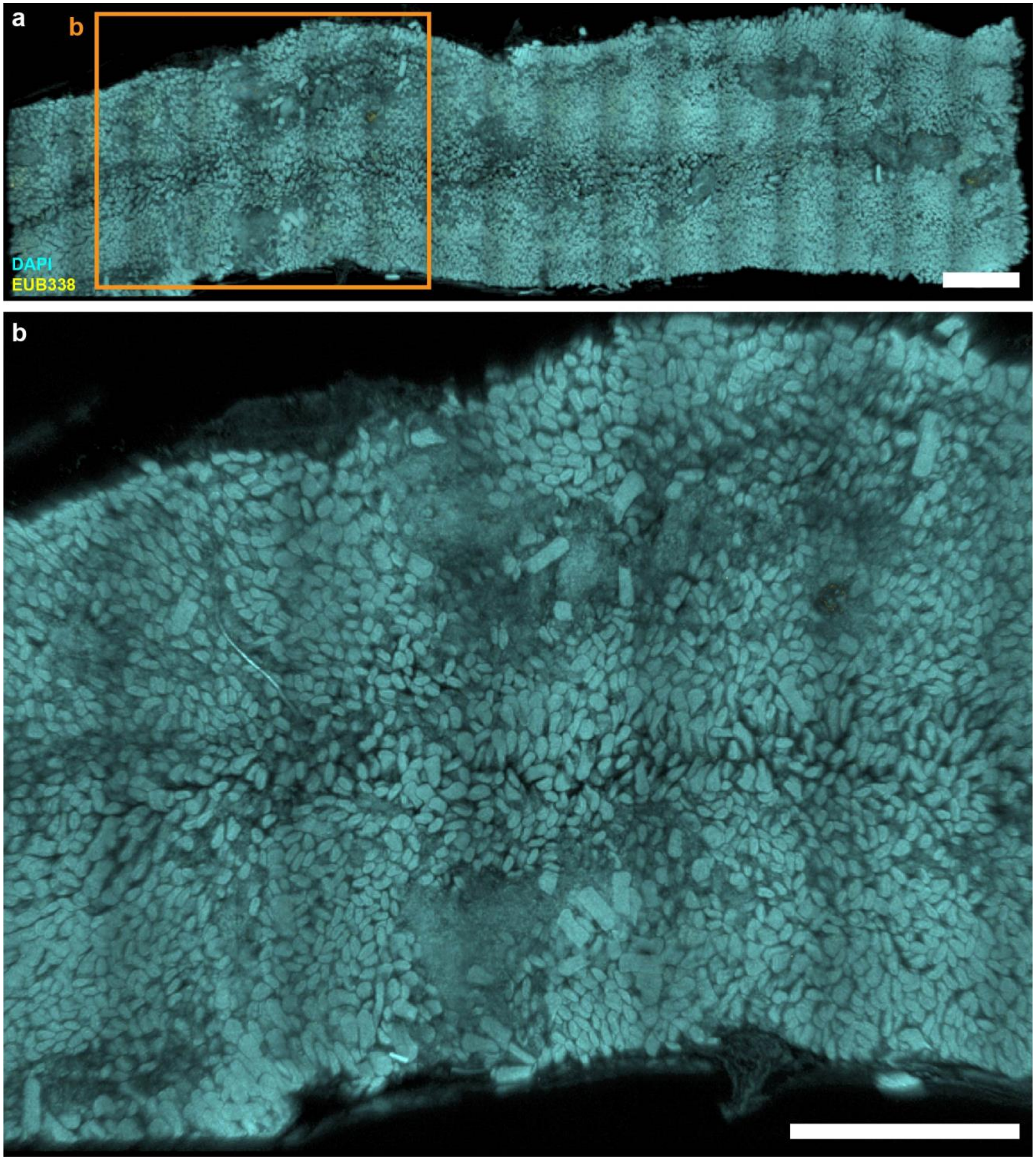

Supplementary Figure 19. Large-scale low-magnification fluorescence imaging of empty jejunum from MAL+EC&BAC mouse on day 31 of the experiment. (a) Large-scale low-magnification tile scan of whole empty jejunum segment without digesta showing DAPI staining of epithelial surface (cyan) and HCR v3.0 staining of total bacteria (yellow). (b) Enlarged portion of the image shown in a (orange rectangle). In this part of the sample, villus loss is clearly visible. All scale bars 2 mm.

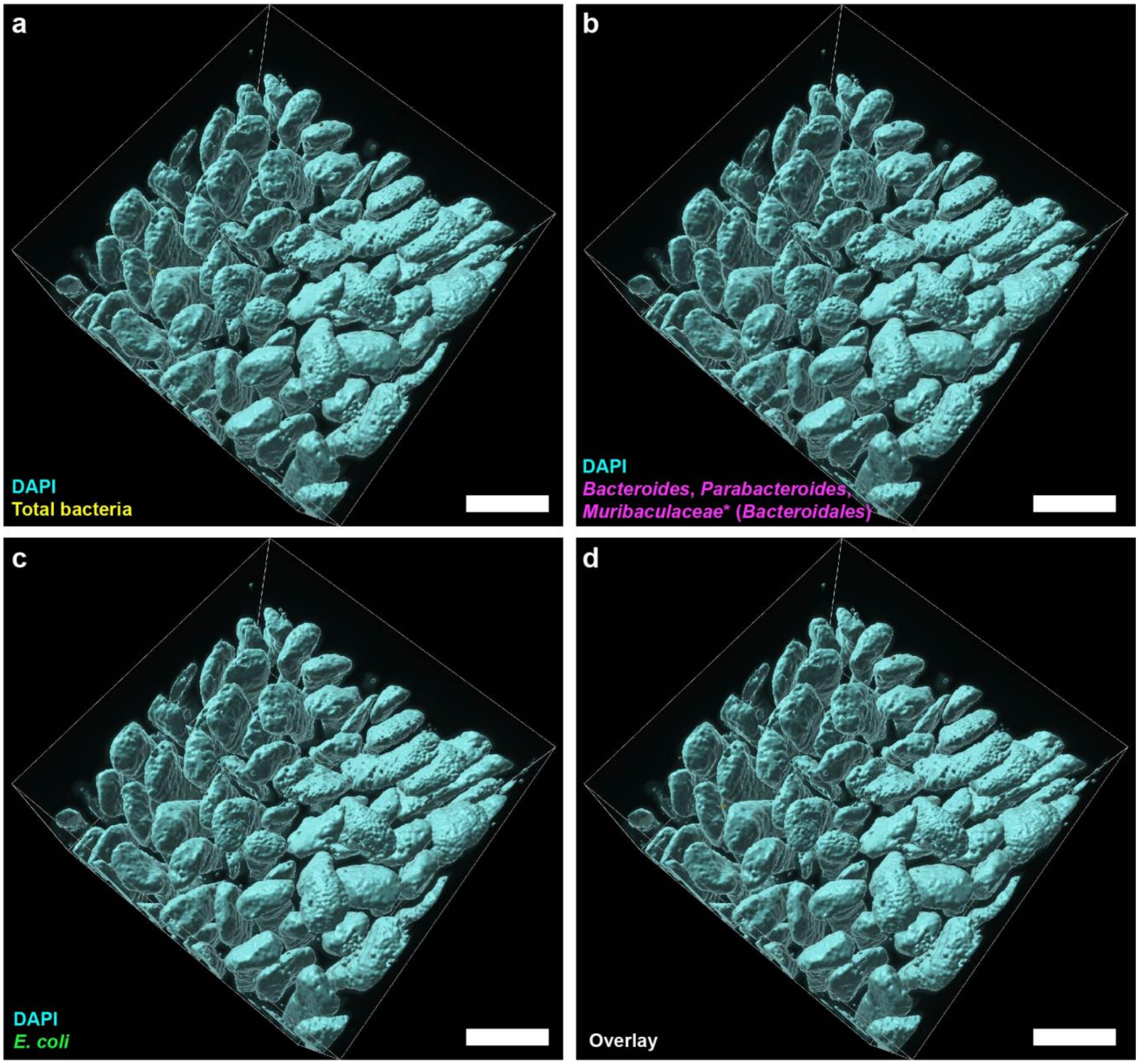

Supplementary Figure 20. High-magnification 3D fluorescence imaging of bacteria after 1-h fast in an empty jejunum of MAL+PBS mouse on day 29 of the experiment. (a-d) 3D rendering of the segmented and filtered surfaces displaying DAPI staining of epithelium (cyan) and (a) HCR v3.0 staining for total bacteria (yellow), (b) HCR v3.0 staining for *Bacteroidales*, (c) HCR v3.0 staining for *E. coli* (green), and (d) overlay with all groups of bacteria. No significant number of bacteria was detected in this field of view. All scale bars 200 µm.

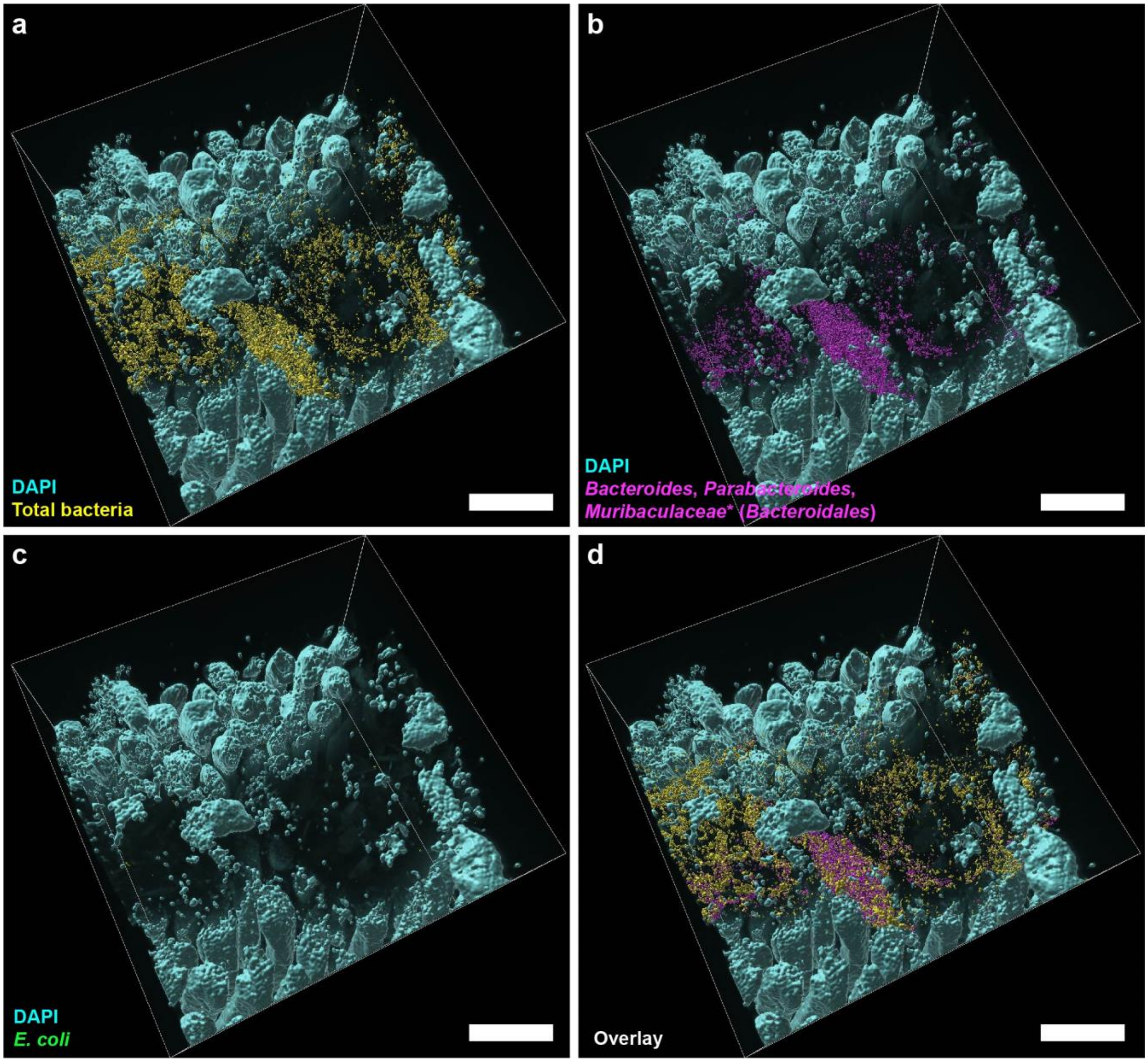

Supplementary Figure 21. High-magnification 3D fluorescence imaging of bacteria after 1-h fast in an empty jejunum of MAL+BAC mouse on day 29 of the experiment. (a-d) 3D rendering of the segmented and filtered surfaces displaying DAPI staining of epithelium (cyan) and (a) HCR v3.0 staining for total bacteria (yellow), (b) HCR v3.0 staining for *Bacteroidales*, (c) HCR v3.0 staining for *E. coli* (green), and (d) overlay all groups of bacteria. A large surface aggregate with abundant bacteria is visible in this field of view. All scale bars 200 µm.

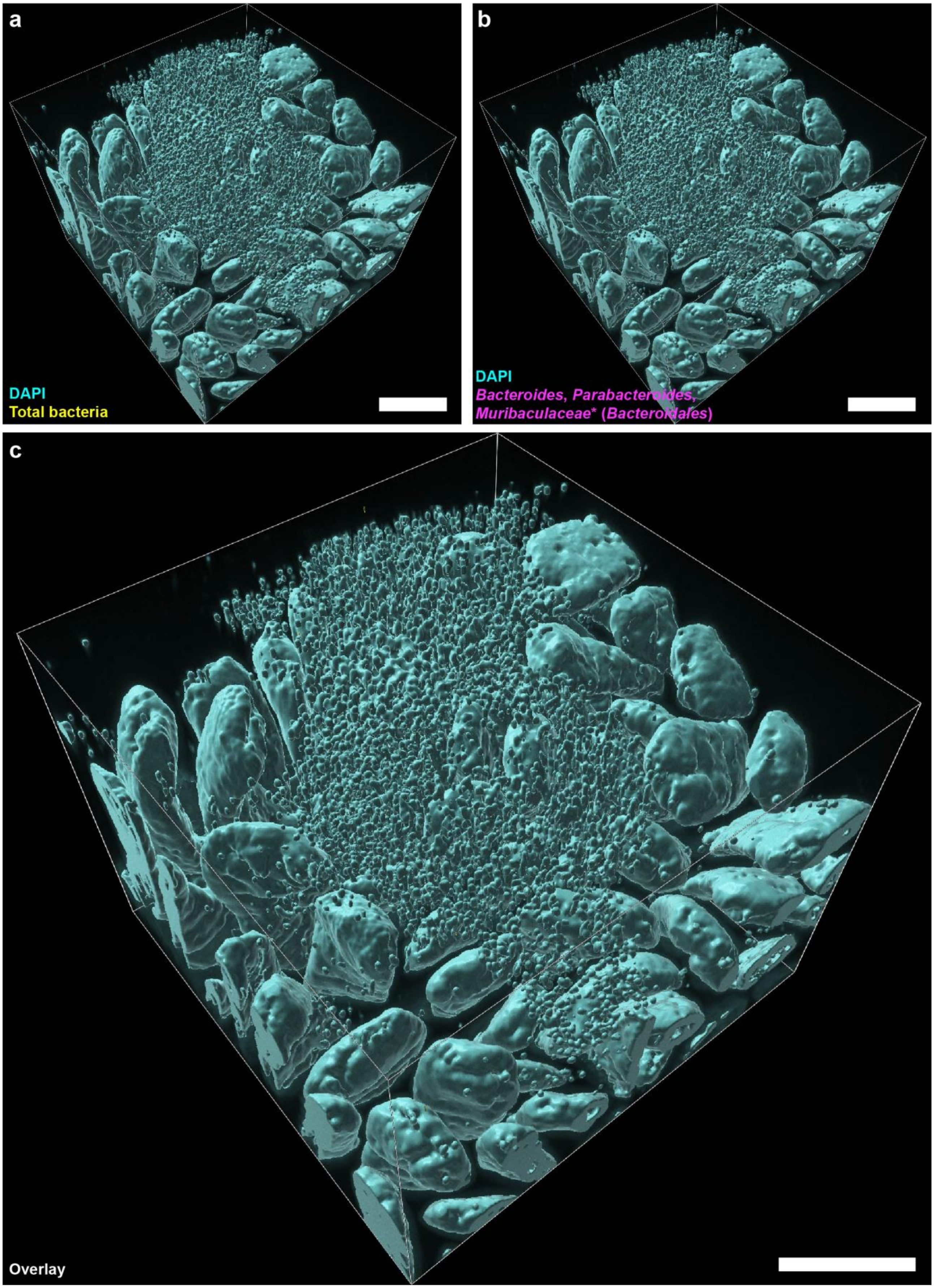

Supplementary Figure 22. High-magnification 3D fluorescence imaging of bacteria after 1-h fast in an empty jejunum of MAL+EC mouse on day 28 of the experiment. (a-c) 3D rendering of the segmented and filtered surfaces displaying DAPI staining of epithelium (cyan) and (a) HCR v3.0 staining for total bacteria (yellow), (b) HCR v3.0 staining for *Bacteroidales*, and (c) overlay with all groups of bacteria. In this field of view, no appreciable number of bacteria and no *E. coli* was detected, but a cluster of abundant free mammalian nuclei is visible. All scale bars 200 µm.

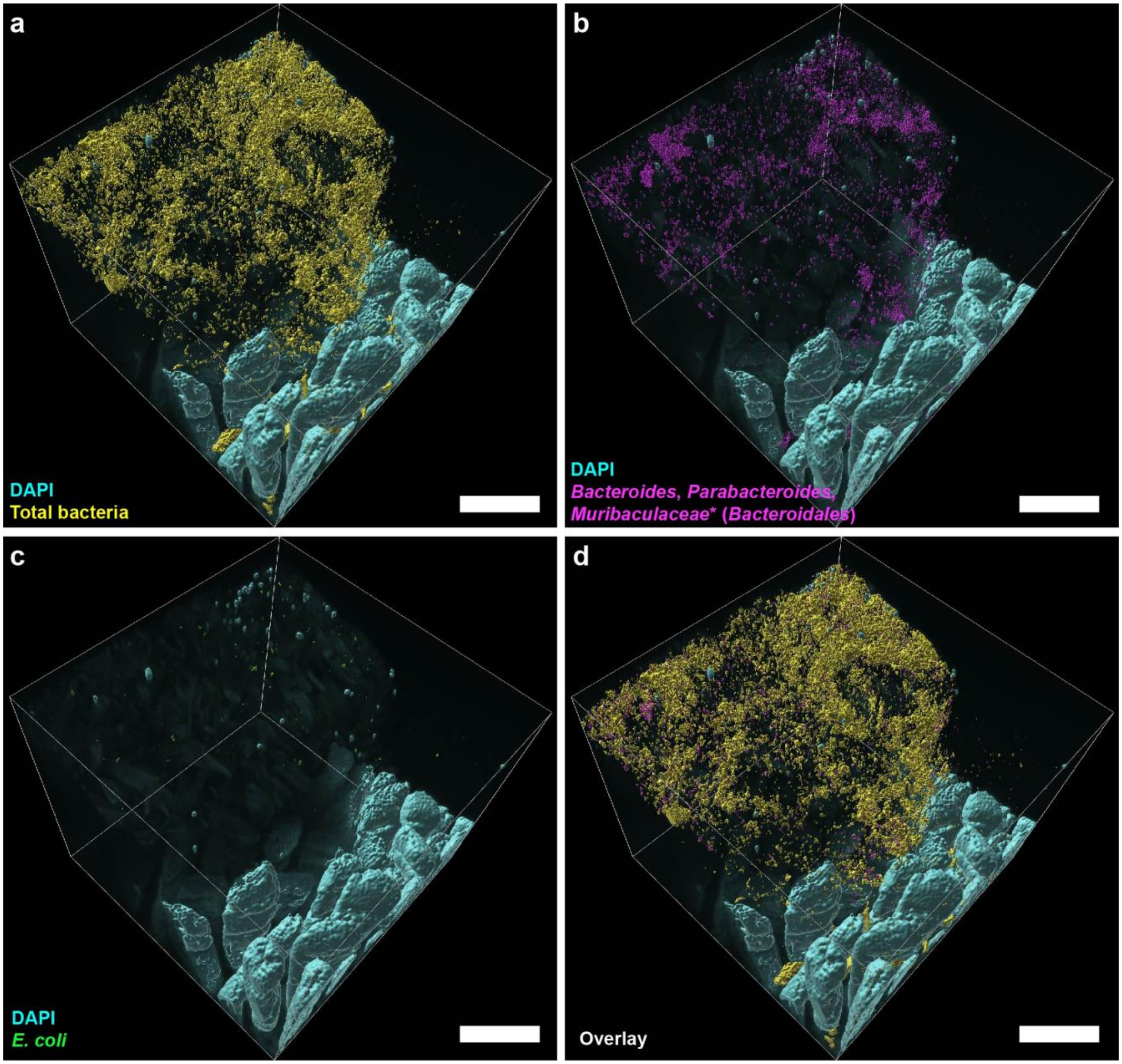

Supplementary Figure 23. High-magnification 3D fluorescence imaging of bacteria after 1-h fast in an empty jejunum of MAL+EC&BAC mouse on day 28 of the experiment. (a-d) 3D rendering of the segmented and filtered surfaces displaying DAPI staining of epithelium (cyan) and (a) HCR v3.0 staining for total bacteria (yellow), (b) HCR v3.0 staining for *Bacteroidales*, (c) HCR v3.0 staining for *E. coli* (green), and (d) overlay all groups of bacteria. A large surface aggregate with abundant bacteria is visible in this field of view. Next to this large surface aggregate, abundant bacteria in between the villi are also visible. All scale bars 200 µm.

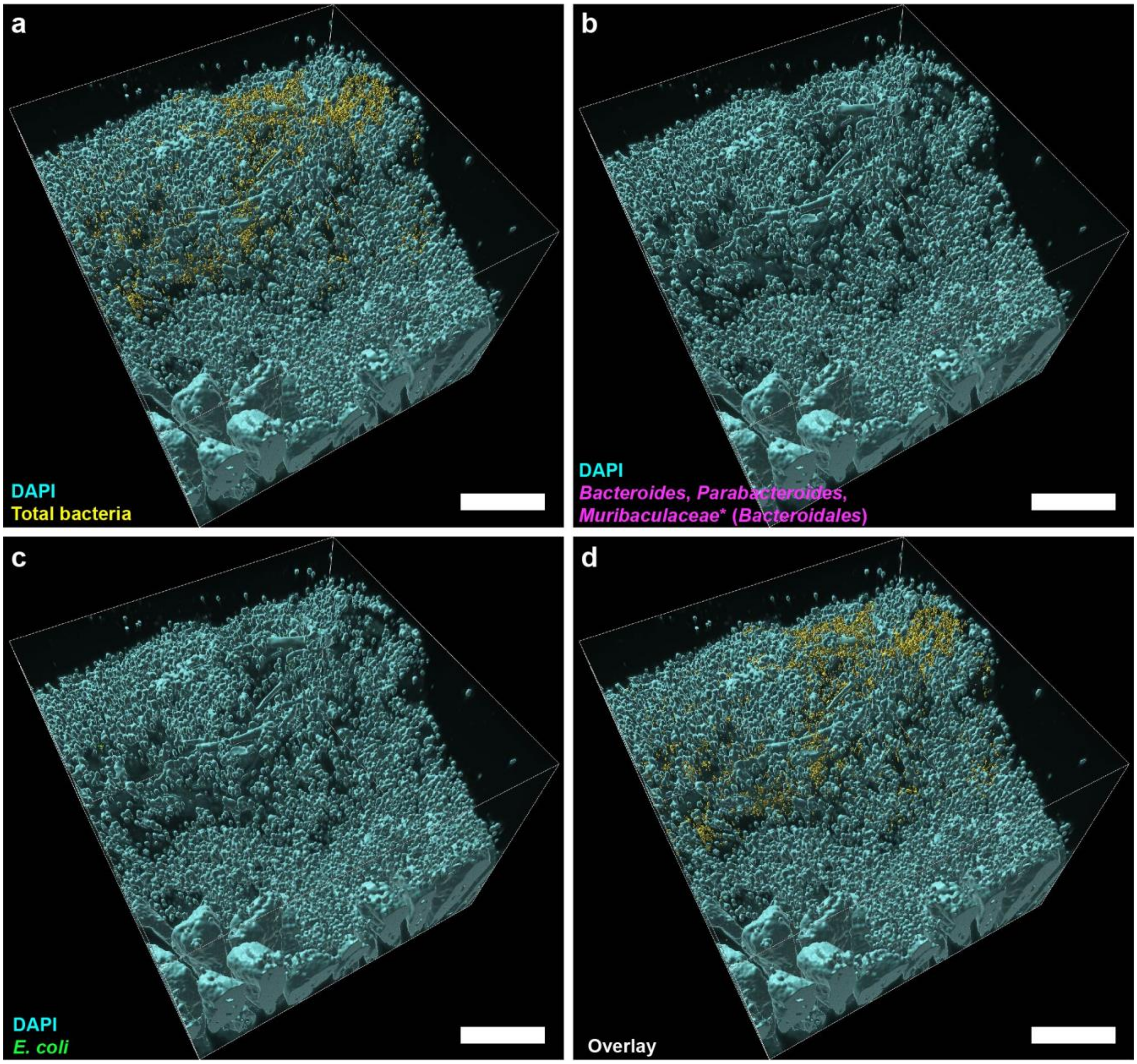

Supplementary Figure 24. High-magnification 3D fluorescence imaging of bacteria after 1-h fast in an empty jejunum of MAL+EC&BAC mouse on day 31 of the experiment. (a-d) 3D rendering of the segmented and filtered surfaces displaying DAPI staining of epithelium (cyan) and (a) HCR v3.0 staining for total bacteria (yellow), (b) HCR v3.0 staining for *Bacteroidales*, (c) HCR v3.0 staining for *E. coli* (green), and (d) overlay all groups of bacteria. A large surface aggregate with bacteria and free mammalian nuclei is visible in this field of view. All scale bars 200 µm.

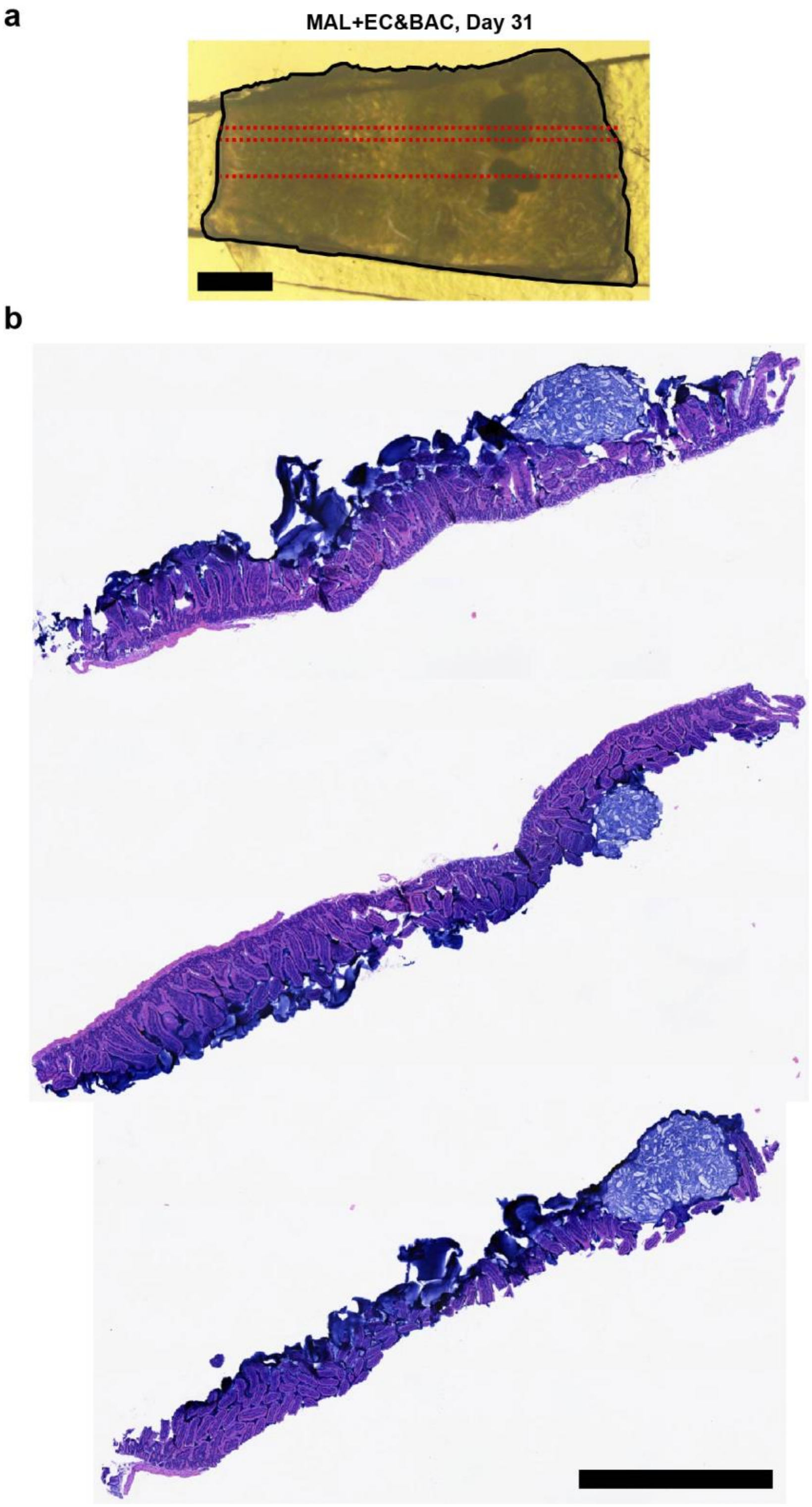

Supplementary Figure 25. 2D imaging of H&E-stained thin sections of a hydrogel-tissue hybrid with large surface aggregates from MAL+EC&BAC mouse. (a) Photo of a hydrogel-tissue hybrid showing the location of thin sections. (b) 2D images of H&E-stained thin sections. The surface gel was stained blue. All scale bars 1 mm.

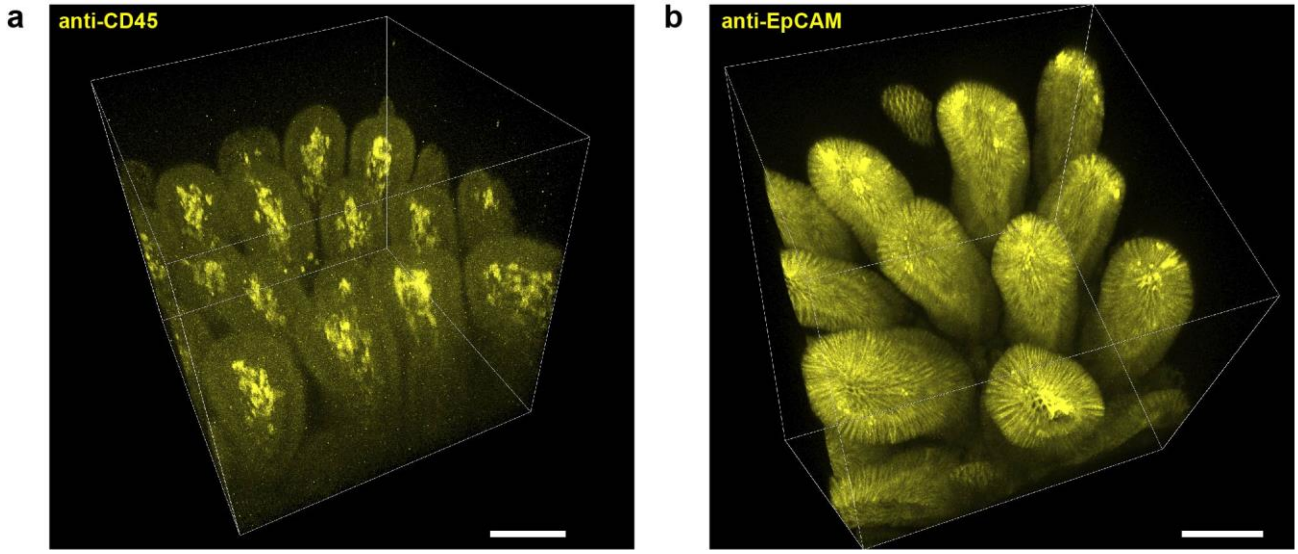

Supplementary Figure 26. Antibody validation in small intestinal hydrogel-tissue hybrids from an SPF mouse. (a) Anti-CD45 staining of immune cells in the core of the villi. (b) Anti-EpCAM staining of epithelial cells on the surface of the villi. All scale bars 100 µm.

Supplementary Tables

Supplementary Table 1. Compositions of malnourished (MAL) and complete control (COM) diets. Diet compositions are identical as previously described[4] except that food dyes have been omitted.

	Control Diet		Malnourished Diet	
**Ingredients**	**grams**	**kcal**	**grams**	**kcal**
Casein	200	800	71	284
L-Cystine	3	12	1.07	4
Corn Starch	346	1384	557	2228
Maltodextrin 10	45	180	70	280
Dextrose	250	1000	250	1000
Sucrose	0	0	2.41	10
Cellulose BW200	75	0	75	0
Inulin	25	25	25	25
Soybean Oil	70	630	23.3	210
Mineral Mix S10026	10	0	10	0
Dicalcium Phosphate	13	0	13	0
Calcium Carbonate	5.5	0	5.5	0
Potassium Citrate, 1xH2O	16.5	0	16.5	0
Vitamin Mix V10001	10	40	10	40
Choline Bitartrate	2	0	2	0
Red Dye #40, FD&C	0	0	0	0
Blue Dye #1, FD&C	0	0	0	0
Yellow Dye #5, FD&C	0	0	0	0
**Total**	**1071**	**4071**	**1131.78**	**4081**
**Breakdown**	**grams (%)**	**kcal (%)**	**grams (%)**	**kcal (%)**
Protein	19.0	19.9	6.4	7.1
Carbohydrates	63.1	64.6	80.8	87.8
Fat	6.5	15.5	2.1	5.1
**Total**		**100**		**100**
**kcal/gm**	**3.80**		**3.61**	

Supplementary Table 2. Surface-gel and tissue-gel monomer mix chemistries used to embed tissues into a hydrogel matrix.

	Total % and amount	Acrylamide, 40%[a]	Bis-acrylamide, 2%[b]	PFA, 32%[c]	PBS, 10x	UltraPure Water	VA044 thermal initiator, CAS NO. 27776-21-2[d]
Surface-gel-monomer mix chemistries							
A4B.08P4	100%	4%	0.08%	4.05%	NA	NA	0.25 w/v%
	30 mL	3 mL	1.2 mL	3.8 mL	3 mL	19 mL	75 mg
A4B.08P1	100%	4%	0.08%	1.07%	NA	NA	0.25 w/v%
	30 mL	3 mL	1.2 mL	1 mL	3 mL	21.8 mL	75 mg
Tissue-gel-monomer mix chemistries							
A4B0P4	100%	4%	0%	4.05%	NA	NA	0.25 w/v%
	30 mL	3 mL	0 mL	3.8 mL	3 mL	20.2 mL	80 mg
A1B.01P4	100%	1.25%	0.0125%	4%	NA	NA	0.25 w/v%
	32 mL	1 mL	0.2 mL	4 mL	3.2 mL	23.6 mL	80 mg

[a]01697; Sigma-Aldrich, St. Louis, MO, USA
b161-0142; Bio-Rad Laboratories, Hercules, CA, USA
c100504-858; VWR, Randor, PA, USA
[d]011-193365; Fujifilm Wako Chemicals, Osaka, Japan

Supplementary Video and Data Captions

Supplementary Video 1. High-magnification 3D imaging of bacterial colonization of SI mucosa in the empty jejunum of a MAL+EC&BAC mouse. (0:00:00 – 0:00:02) 3D rendering of the fluorescence intensity data. (0:00:02 – 0:00:09). Fluorescence intensity data across z-sections. (0:00:09 – 0:00:09) 3D rendering of the fluorescence intensity data. (0:00:11 – 00:00:18) Transformation of fluorescence intensity data to the segmented surfaces. 0:00:18-0:00:30) 3D rendering of the segmented surfaces. Cyan: DAPI staining of epithelium. Yellow: HCR v3.0 staining of total bacteria. The same image is displayed in Supplementary Video 1 as in Figure 4b.

Supplementary Data 1. 16S rRNA gene amplicon sequencing data presented as the number of reads per taxon per sample. Analysis at different taxonomic levels is provided in separate tabs. Supplementary Data 1 is displayed in Fig. 2a.

Supplementary Data 2. Data for DNA extraction and 16S rRNA gene copy quantification by dPCR. Supplementary Data 2 is displayed in Fig. 2, b and e-g, and Supplementary Fig. 2, a and c.

Supplementary Data 3. Sequences of HCR v2.0 probes, HCR v3.0 probes, and HCR initiators. Regions of HCR v3.0 probes that overlap with the corresponding HCR v2.0 probes are highlighted in bold.

Supplementary Data 4. Imaging, display, segmentation, and filtering metadata.

Supplementary Methods

Validation of the universal degenerate HCR v3.0 probe set

The universal degenerate HCR v3.0 probe set was validated on a proximal colon segment from a CHOW-fed C57BL/6J mouse (Supplementary Fig. 9). The segment was preserved following the protocol for empty jejunum segments from fasted mice (see Methods). The resulting hydrogel-tissue hybrid was permeabilized with lysozyme and cleared with SDS as described in the main text except that clearing duration was extended to 5 days because proximal colon tissue is thicker than jejunum tissue. The cleared hydrogel-tissue hybrid was stained for total bacteria following HCR v3.0 protocol with modifications. Specifically, the hydrogel-tissue hybrid was hybridized with EUB338 HCR v2.0 probe linked to B4 initiator and the universal degenerate HCR v3.0 probe set linked to B2 initiator in 3 mL of probe hybridization buffer for tissues in whole-mount (Molecular Technologies). The concentration of each universal degenerate HCR v3.0 probe was 4 nM; considering that each arm of the split universal HCR v3.0 probe set contains ~10 probes (6 and 12 to be exact), we set EUB338 concentration to 40 nM to avoid out-competition. Hybridized probes were amplified with B4-AlexaFluor546 and B2-AlexaFluor647 amplifier pairs in 1.5 mL of amplification buffer for tissues in whole-mount (Molecular Technologies). After HCR, the hydrogel-tissue hybrid was stained overnight at room temperature (RT) with 5 µg/mL DAPI and 5 µg/mL WGA-AlexaFluor488 in 5 mL of PBS. After overnight infusion in RIMS, the sample was imaged with the 20x CLARITY objective. All imaging and display metadata are provided in Supplementary Data 4. In Supplementary Fig. 9l, signal was defined as voxel fluorescence, and background was defined as average voxel fluorescence of DAPI+ voxels (voxel fluorescence in DAPI channel >200).

Validation of taxon-specific HCR v3.0 probes

Taxon-specific HCR v3.0 probes were validated *in vitro* on two isolates in the bacterial cocktail: *E. coli* and *B. fragilis* (Supplementary Fig. 10). *E. coli* and *B. fragilis* suspensions in PBS were prepared as for bacterial gavage described in the main text. These suspensions were then mixed with 8% PFA in 1:1 ratio and fixed on ice for 1 h. Fixed bacterial cells were spiked into A4B.08P1 surface gel monomer mix at ~$5 \cdot 10^7$ cells/mL density and polymerized into a hydrogel slab; hydrogel embedding protocol for empty jejunum segments from fasted mice was otherwise followed. The resulting hydrogel slabs were permeabilized with lysozyme and cleared with SDS as described in the main text except that clearing duration was reduced to 1 d. Processed *E. coli* and *B. fragilis* gel slabs were stained following the HCR v3.0 protocol (see Methods) except that the slabs were hybridized with *E. coli* and *Bacteroidales* HCR v3.0 probes linked to B5 and B4 initiators, respectively, each at 10 nM concentration in 1 mL of probe hybridization buffer for tissues in whole-mount, and then amplified with B5-AlexaFluor488 and B4-AlexaFluor546 amplifiers in 0.5 mL of amplification buffer for tissues in whole-mount. After HCR, the gel slabs were stained overnight with 1 µM TO-PRO-3 Iodide DNA stain (T3605; ThermoFisher Scientific) in PBS and then imaged with 20x water immersion objective. Imaging and display settings are provided in Supplementary Data 4.

Comparison of taxon-specific HCR v2.0 and HCR v3.0 probe sets

Taxon-specific HCR v2.0 and HCR v3.0 probes have been compared *in vitro* on one of the *E. coli* isolates only (Supplementary Fig. 11). *E. coli* gel slab was prepared and processed as described above. *E. coli* gel slab was then stained following HCR v3.0 protocol except that it was hybridized with CFB560b-B5 HCR v2.0 and *Bacteroidales*-B3 HCR v3.0 probes, each at 10 nM in 1 mL of probe hybridization buffer for tissues in whole-mount, and then amplified with B5-AlexaFluor488 and B3-AlexaFluor594 amplifiers in 0.5 mL of amplification buffer for tissues in whole-mount. After HCR tagging of bacteria, the hydrogels were counterstained with 5 µg/mL of DAPI overnight and imaged with 20x water immersion objective. Imaging, display, and segmentation settings are provided in Supplementary Data 4. In Supplementary Fig 11, b and c, the signal was defined as average voxel fluorescence in DAPI+ surfaces obtained by DAPI segmentation, and background was defined as the most frequent voxel fluorescence value in the entire image (that is, the peak of voxel fluorescence histogram).

Comparison of A1B.01P4 and A4B0P4 tissue gel formulations

The effect of tissue gel formulation on antibody staining was compared on jejunum segments from a CHOW-fed C57BL/6J mouse (Supplementary Fig. 12). Two segments were preserved following the protocol for empty jejunum segments from non-fasted mice with modifications. Specifically, transcardial perfusion as well as PFA in the surface gel monomer mix were omitted, and one segment was embedded into A4B0P4 tissue gel with 3 h polymerization duration. The other segment was embedded into A1B.01P4 tissue gel with 5 h polymerization duration (see Supplementary Data 4 for monomer mix compositions). The hydrogel-tissue hybrids were then permeabilized with lysozyme and cleared with SDS as described in the main text except that clearing duration was extended to 5 d. Clearing progress was monitored daily by measuring hydrogel-tissue hybrid absorbance with a portable spectrophotometer (80-2116-30; Harvard Bioscience, Holliston, MA, USA) (Supplementary Fig. 12d). After clearing, net protein loss was quantified with the microBCA Protein Assay Kit (23235, ThermoFisher Scientific) following manufacturer's instructions (Supplementary Fig. 12c). Processed hydrogel-tissue hybrids were stained with 5 µg/mL anti-CD45 antibody and 5 µg/mL DAPI as described in the main text except that staining duration was reduced to 24 h. Finally, stained samples were mounted in RIMS and imaged with 20x CLARITY objective (imaging metadata is provided in Supplementary Data 4). One z-stack was acquired for each condition, for a total of 2 z-stacks.

Antibody penetration (Supplementary Fig. 12b) was quantified in FIJI. Briefly, a region of interest (ROI) was first drawn to mark the tip of a villus so that the z-distance from the villus tip could later be quantified. Then, at various planes along the villus, four ROIs were selected: three marking immune cells in the core of the villus (signal) and one marking epithelial cells (background). Average ROI fluorescence was calculated in FIJI, and the results were exported for further analysis in Python. In Python, the signal-background ratio was calculated as the ratio of average fluorescence in signal ROI to average fluorescence in background ROI at the same z-plane. Each data point in Supplementary Fig. 12b represents signal-background ratio averaged over three signal ROIs at the same plane.

Histopathology

A portion of a single hydrogel-tissue hybrid with large surface aggregates from MAL+EC&BAC mouse euthanized on day 31 (Supplementary Fig. 25a) was sent to IDEXX Laboratories (Westbrook, ME, USA) for paraffin embedding, sectioning, hematoxylin and eosin (H&E) staining, imaging, and histopathology analysis. Paraffin blocks were cut into 4 µm sections. Three sections were evaluated by the pathologist (Supplementary Fig. 25b). Surface gel was stained blue by H&E staining (Supplementary Fig. 25b).

Supplementary References

1 Livak, K. J. & Schmittgen, T. D. Analysis of relative gene expression data using real-time quantitative PCR and the 2-ΔΔCT method. *Methods* **25**, 402-408, (2001).

2 Barlow, J. T., Bogatyrev, S. R. & Ismagilov, R. F. A quantitative sequencing framework for absolute abundance measurements of mucosal and lumenal microbial communities. *Nat. Comm.* **11**, 1-13, (2020).

3 Choi, H. M. T. *et al.* Third-generation in situ hybridization chain reaction: Multiplexed, quantitative, sensitive, versatile, robust. *Development* **145**, 1-10, (2018).

4 Brown, E. M. *et al.* Diet and specific microbial exposure trigger features of environmental enteropathy in a novel murine model. *Nat. Commun.* **6**, 1-16, (2015).

Author Contributions

Roberta Poceviciute (RP)

1. **Idea generation.** Conceived the project with RFI. Conceived the idea of imaging bacteria in the empty intestinal segments of malnourished mice gavaged with bacteria. Conceived the idea of imaging bacteria after 1 h fast. Wrote or contributed to writing grant proposals to fund the project.
2. **Preliminary experiments.** Performed preliminary 16S rRNA gene amplicon sequencing (in collaboration with OMP), 16S rRNA gene copy quantification by dPCR, and imaging (in collaboration with OMP). Supervised preliminary machine-learning based image analysis (performed by HT). Used preliminary data to refine the project.
3. **Method development (HCR v3.0 probe design and validation, hydrogel chemistry optimization).** Starting with the 52 bp region that overlaps with EUB338 binding site (selected by Molecular Technologies), designed the universal degenerate HCR v3.0 probe set. Validated HCR v3.0 probes. Screened different hydrogel chemistries for optimal transport properties.
4. **Data acquisition.** Set up all animal experiments (tail cup experiment was set up in collaboration with SB). Collected and preserved samples for imaging. Collected samples for host gene expression analysis by RTqPCR and 16S rRNA gene analysis by dPCR and sequencing with SRB. Extracted RNA for RTqPCR in collaboration with AER. Acquired all imaging data.
5. **Data analysis.** Analyzed RT-qPCR and dPCR data, processed 16S rRNA gene amplicon sequencing, and imaging data. Conceived image segmentation and filtering pipeline.
6. **Figure generation.** Created all figures in the main text and the SI.
7. **Outline writing.** Conceived and wrote outlines.
8. **Manuscript writing.** Wrote and edited the manuscript.

Said R. Bogatyrev

1. **Data acquisition.** Set up tail cup experiment with RP. Collected samples for host gene expression analysis by RTqPCR and 16S rRNA gene analysis by dPCR and sequencing with RP.

Anna E. Romano

1. **Data acquisition.** Extracted RNA with RP and quantified host gene expression by RTqPCR (Supplementary Fig. 1, b and c). Extracted DNA and quantified 16S rRNA gene copy load (Fig. 2, f and g). Prepared 16S rRNA gene amplicon library for sequencing (Fig. 2a).

Amanda H. Dilmore

1. **Data acquisition.** Extracted DNA for 16S rRNA gene amplicon sequencing and 16S rRNA gene copy quantification (Fig. 2, a-e). Quantified 16S rRNA gene copies by dPCR (Fig. 2, b-e, and Supplementary Fig. 2).

Octavio Mondragón-Palomino

1. **Preliminary experiments.** Performed preliminary 16S rRNA gene amplicon sequencing (in collaboration with RP) and 3D imaging of tissue samples from the small intestines of mice in the environmental enteropathy model (in collaboration with RP). Contributed practical and technical guidance on spectral confocal imaging of clarified tissues.

Heli Takko

1. **Preliminary experiments.** Performed preliminary machine-learning based image analysis (supervised by RP).

Ojas Pradhan

1. **Data acquisition**. Managed histopathology data acquisition (Supplementary Fig. 25)

Rustem F. Ismagilov

1. **Idea generation.** Conceived the project with RP.
2. **Data acquisition.** Provided feedback on experimental design.
3. **Funding.** Secured funding for the project.
4. **Data analysis.** Provided feedback on data analysis.
5. **Outline writing.** Provided feedback on outlines written by RP.
6. **Manuscript writing.** Edited the manuscript written by RP.
